# Clinical utility of circulating tumor DNA for early detection of recurrence after curative hepatectomy in patients with colorectal cancer with liver metastases: A prospective observational study protocol (CASSIOPEIA)

**DOI:** 10.1371/journal.pone.0335591

**Published:** 2025-11-20

**Authors:** Akira Inoue, Yoshihiro Morimoto, Yujiro Nishizawa, Masahiro Hashimoto, Yuki Ozato, Kenta Furukawa, Masashi Hirota, Yasuhiro Miyazaki, Akira Tomokuni, Masaaki Motoori, Kazumasa Fujitani

**Affiliations:** Department of Gastroenterological Surgery, Osaka General Medical Center, Osaka, Japan; King Saud University, SAUDI ARABIA

## Abstract

Colorectal cancer (CRC) with liver-only metastases remains a significant clinical challenge owing to its high recurrence rate, even after curative hepatectomy. Despite advancements in surgical techniques and systemic therapies, identifying patients at elevated risk of recurrence is crucial for optimizing long-term outcomes. Circulating tumor DNA (ctDNA) is a biomarker for minimal residual disease and recurrence risk assessment. We hypothesize that plasma-only ctDNA assays using the Plasma-Safe-SeqS platform can enable early recurrence detection and guide postoperative management, including adjuvant chemotherapy. This single-center, prospective observational study will enroll 10 patients with histologically confirmed CRC and liver-only metastases undergoing curative hepatectomy. Plasma samples are collected preoperatively and at predefined intervals postoperatively (4, 12, 24, 36, and 48 weeks) to monitor ctDNA levels using a highly sensitive 14-gene panel designed to detect tumor-specific mutations. Mutant allele frequencies as low as 0.1% are detected using the Plasma-Safe-SeqS platform. The primary endpoint is the interval between ctDNA detection and clinically confirmed recurrence. The secondary endpoints include mutation concordance between pre- and postoperative samples, 3-year disease-free survival, 5-year overall survival, and correlations with clinicopathological features. Kaplan–Meier estimates and Cox proportional hazards models are used for statistical analyses. To our knowledge, this is the first study to utilize a 14-gene panel with Plasma-Safe-SeqS technology for ctDNA analysis in patients with CRC liver metastases following curative hepatectomy. By eliminating the need for tumor tissue, this plasma-only approach simplifies diagnostics and mitigates logistical barriers, including prolonged turnaround times associated with tumor-informed assays. We aim to validate ctDNA as a reliable biomarker for early recurrence detection. Furthermore, we may provide valuable insights into optimizing adjuvant chemotherapy strategies by identifying high-risk patients who may benefit from treatment while sparing low-risk patients from unnecessary toxicity. These findings may advance personalized postoperative care and enhance long-term outcomes in this patient population.

Trial registration

The study is registered in accordance with the International Committee of Medical Journal Editors (ICMJE) guidelines under the University Hospital Medical Information Network Clinical Trials Registry (UMIN-CTR). The registry name is UMIN-CTR. The registration number is UMIN000057128. Trial details are available upon request.

## Introduction

Colorectal cancer (CRC) with liver metastases presents a significant clinical challenge as a leading cause of cancer-related mortality worldwide [1]. Liver metastases occur in approximately 25%–50% of CRC cases, and recurrence rates remain alarmingly high at 50%–70% despite curative-intent surgery [2]. Accurate prognosis and stratification of recurrence risk are critical for improving patient outcomes. However, existing prognostic models, which rely on clinicopathological factors, such as tumor size, number of metastases, and *RAS* mutation status, are insufficient to capture the complex biology underlying recurrence. The role of adjuvant chemotherapy (ACT) in this population remains controversial. Although ACT has demonstrated potential benefits in certain subsets of patients, its efficacy is inconsistent, and ACT administration is usually not guided by the use of robust predictive biomarkers [3–5]. This underscores the urgent need for innovative tools to refine postoperative risk stratification and optimize ACT strategies to improve survival outcomes.

Circulating tumor DNA (ctDNA) is a non-invasive biomarker for minimal residual disease and relapse detection after curative treatments of solid tumors. An earlier pioneering study by Tie et al. demonstrated the clinical utility of ctDNA by showing its role in detecting minimal residual disease and predicting recurrence in stage II colon cancer [6]. In addition, the GALAXY trial, part of the CIRCULATE-Japan platform, has been pivotal in establishing the clinical utility of ctDNA as a prognostic biomarker in CRC [7,8]. By employing tumor-informed ctDNA assays, GALAXY showed that postoperative ctDNA positivity is strongly associated with recurrence risk and guides ACT administration. This trial provided compelling evidence that ctDNA can stratify patients by recurrence risk more effectively than conventional clinicopathological factors. Using data from the GALAXY study, Kataoka et al. demonstrated that ctDNA positivity correlates with inferior disease-free survival (DFS) in patients with resected colorectal liver metastases, further emphasizing its role in guiding ACT [9]. These studies employed tumor-informed approaches, which, although highly effective, require tumor tissue for assay customization. This requirement can delay clinical decision-making and restrict their applicability in some clinical settings.

To address the limitations of tumor-informed methods, we have initiated the CASSIOPEIA study, a prospective observational trial that employs a plasma-only ctDNA assay. Unlike tumor-informed assays, this approach eliminates the need for tumor tissue, streamlining the diagnostic process, reducing turnaround time, and maintaining high sensitivity and specificity. Utilizing the advanced Plasma-Safe-SeqS next-generation sequencing method, this assay detects mutations at allele frequencies as low as 0.04% for 10,000 genome equivalents (GEs) of DNA input, making it particularly advantageous in clinical scenarios requiring rapid decision-making or where tumor tissue is unavailable [10]. This level of sensitivity is comparable to that of tumor-informed assays while offering a more practical and clinically implementable solution. To date, the Plasma-Safe-SeqS platform in a fully tumor-naïve design has not been prospectively validated in published studies of CRC. However, the platform has been incorporated into two prospective clinical trials. One is the ongoing ASCOLT trial (NCT00565708), a multicenter randomized study evaluating adjuvant aspirin in patients with resected high-risk CRC, where ctDNA analysis using Plasma-Safe-SeqS has been presented in a conference abstract [11]. The other is a multicenter prospective study in patients with Stage II CRC, presented at the 2022 ASCO Annual Meeting [12], which demonstrated the feasibility of Plasma-Safe-SeqS for minimal residual disease detection after curative resection. However, full peer-reviewed results from this study are not yet available. To the best of our knowledge, our study is the first to use a peer-reviewed protocol to evaluate this platform in patients with CRC liver metastases and aims to generate foundational feasibility data on its clinical utility in a tumor-naïve application.

The CASSIOPEIA study is based on the hypothesis that plasma-only ctDNA assays can reliably detect recurrence earlier than standard surveillance in patients with CRC with liver-only metastases following curative hepatectomy, offering critical insights into postoperative management. Specifically, we propose that this approach enables early identification of high-risk patients and facilitates tailored surveillance and treatment strategies, overcoming the logistical and operational challenges associated with tumor-informed methods. This prospective, exploratory proof-of-concept protocol is designed to generate the foundational data necessary to plan a subsequent large clinical study.

The CASSIOPEIA study aims to evaluate the efficacy of plasma-only ctDNA assays for detecting recurrence early in patients with CRC and liver-only metastases post-curative resection. By eliminating the need for tumor tissue, this approach simplifies diagnostic workflows and minimizes turnaround time, while maintaining high sensitivity. Focusing on a homogeneous cohort of liver-only metastases ensures robust evaluation of ctDNA prognostic value. This streamlined and scalable method is expected to bridge the gap between advanced molecular diagnostics and routine clinical practice, addressing key challenges identified in previous studies.

## Materials and methods

### Study design

**Fig 1:**
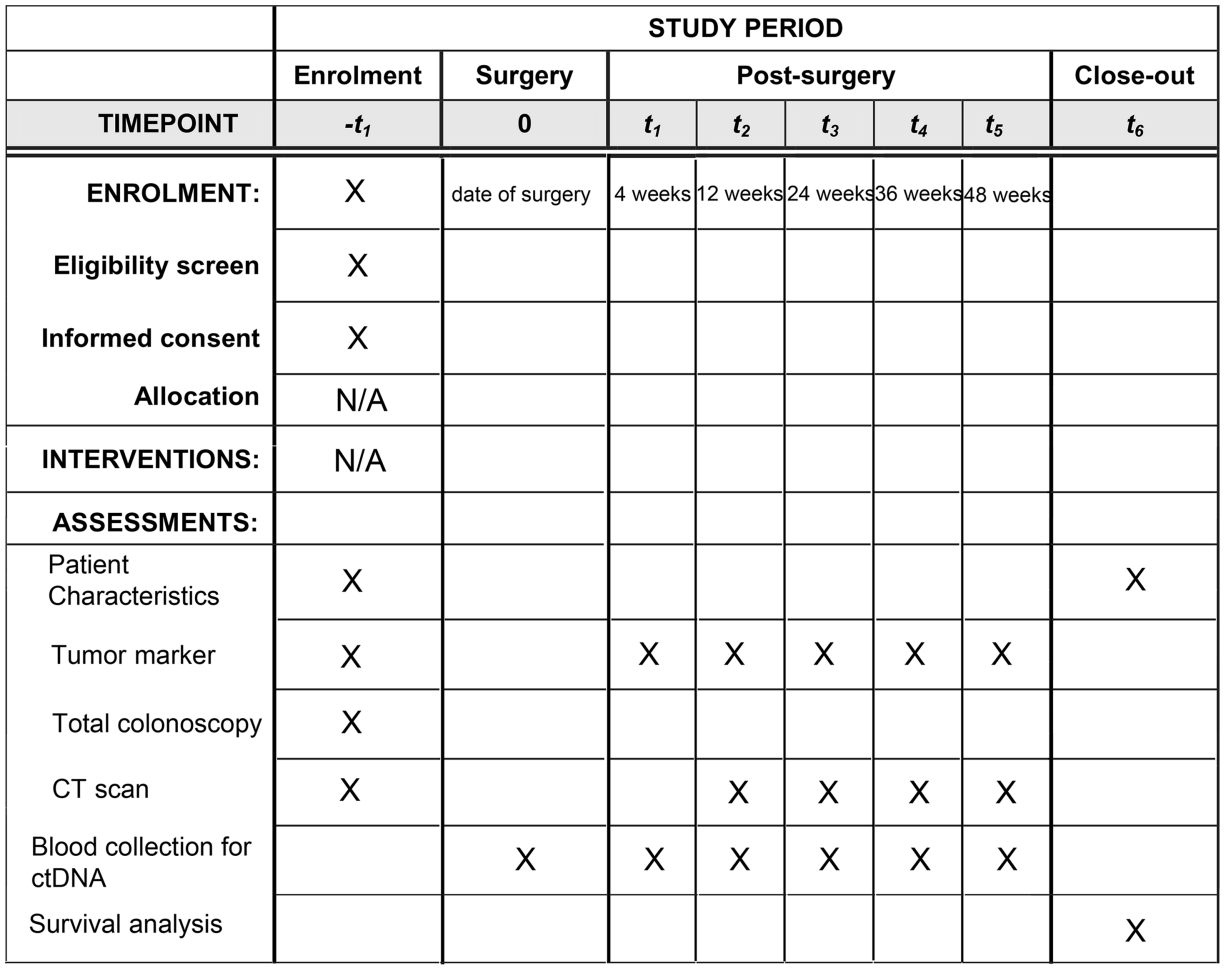
SPIRIT schedule of enrolment, interventions, and assessments for the CASSIOPEIA study.

**Fig 2:**
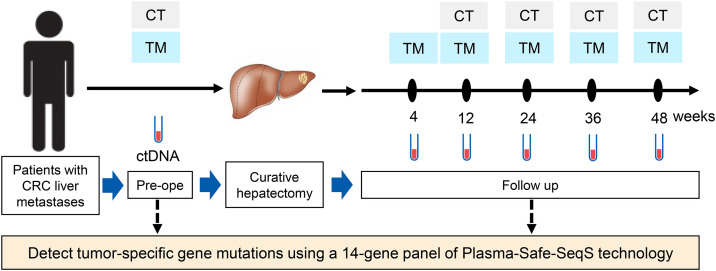
Study design and follow-up protocol for the CASSIOPEIA study. This figure outlines the integration of ctDNA testing into clinical follow-up to assess recurrence dynamics and survival outcomes. The study enrolls patients with CRC liver metastases undergoing curative hepatectomy. Plasma samples for circulating tumor DNA (ctDNA) analysis are collected preoperatively and at predefined intervals postoperatively (4, 12, 24, 36, and 48 weeks). Tumor-related gene mutations are detected using the Plasma-Safe-SeqS gene panel. Routine computed tomography (CT) imaging and tumor marker (TM) evaluations are conducted in parallel with ctDNA testing to monitor recurrence. Abbreviations: CRC, Colorectal cancer; CT, Computed tomography; ctDNA, Circulating tumor DNA; TM, Tumor marker.

The CASSIOPEIA study is a single-center, prospective observational trial designed to evaluate the clinical utility of plasma-only ctDNA assays for detecting early recurrence in patients with CRC and liver-only metastases undergoing curative hepatectomy. A SPIRIT schedule and overview of the study design can be found in Figs 1 and 2.

### Recruitment and study timeline

Patient recruitment began on December 1, 2024, and is expected to continue until December 1, 2025, with a total of 10 patients planned for enrollment. The follow-up period extends for 5 years postoperatively, resulting in a final follow-up date of December 1, 2030.

• Recruitment period: December 1, 2024–December 1, 2025
• Follow up and data collection period: December 1, 2024–December 1, 2030
• Final analysis and results expected: By December 2030

As of the submission of this manuscript, patient recruitment and data collection are currently ongoing, and no final analyses have been conducted.

Plasma samples are collected preoperatively and at predefined postoperative intervals (4, 12, 24, 36, and 48 weeks). The study plans to enroll 10 patients. Given the small sample size, no formal power calculation or hypothesis testing is planned. This study is conducted based on a prospective exploratory, proof-of-concept protocol to explore ctDNA dynamics, survival outcomes, and assay implementation. The results generate sufficient pilot data and inform the design of future larger-scale, statistically powered multicenter studies. Eligibility criteria include patients with histopathological confirmed CRC and liver-only metastases planned for curative hepatectomy. To confirm the absence of extrahepatic disease, all patients undergo contrast-enhanced CT scans of the chest, abdomen, and pelvis, along with abdominal ultrasonography, prior to enrollment. Additional imaging, such as liver MRI or PET-CT, may be performed as clinically indicated. Participants must meet specific conditions, such as an Eastern Cooperative Oncology Group performance status of 0 or 1, and provide informed consent. Exclusion criteria include active double cancers, pregnancy, or conditions deemed unsuitable by the attending physician. The lower age limit is set at 20 years in accordance with the study protocol. Patients with CRC younger than 20 years are extremely rare and often present with hereditary or biologically distinct features. To maintain the clinical homogeneity of the study cohort and ensure reproducibility and reliability of ctDNA dynamics, such cases are excluded. Detailed eligibility and exclusion criteria are outlined in Table 1. A homogeneous cohort of patients with liver-only metastases ensures a robust evaluation of ctDNA dynamics and recurrence prediction.

**Table 1:** Eligibility and exclusion criteria for the CASSIOPEIA study.

Eligibility criteria
1. **Histological Diagnosis**: Histopathologically confirmed adenocarcinoma of colorectal cancer. 2. **Primary Tumor Location and Resection**: The primary tumor is located in the colon (cecum, colon, sigmoid rectum) or rectum and has been resected. Appendiceal and anal canal cancers are excluded. 3. **Liver Metastases**: No distant metastases other than liver metastases, and curative hepatectomy is planned for the first time. Patients with either synchronous or metachronous liver metastases are eligible if no extrahepatic disease is detected and curative hepatectomy is feasible. Liver metastases detected within 6 to 12 months after colorectal resection are classified as synchronous and included. Cases involving systemic chemotherapy enabling curative liver resection (conversion therapy) are also eligible. 4. **Age**: 20 years or older at the time of consent acquisition. 5. **Performance Status**: ECOG PS of 0 or 1. 6. **Informed Consent**: Written informed consent for study participation was obtained from the patient.
**Exclusion Criteria**
1. **Active Double Cancer**: Active overlapping cancers at the time of hepatectomy. Exceptions include the following: (a) relapse-free period of more than 5 years, (b) localized cancers cured through local treatment (e.g., basal or squamous cell carcinoma of the skin, superficial bladder cancer, cervical cancer, ductal carcinoma in situ, or mucosal carcinoma), or (c) non-metastatic prostate cancer not requiring systemic treatment. 2. **Pregnancy or Breastfeeding**: Pregnancy or lactation at the time of the study. 3. **Serious Complications**: Patients with severe comorbidities that could affect the safety or proper implementation of the study. 4. **Viral Infections**: Positive for HBs antigen, HCV antibody, or HIV antibody. 5. **Medical Inappropriateness**: Deemed unsuitable for the study by the attending physician.

This table outlines the eligibility and exclusion criteria for participants in the CASSIOPEIA study. The **Eligibility Criteria** section specifies requirements for inclusion in the study. The **Exclusion Criteria** section lists conditions that disqualify individuals from participation.

Abbreviations: ECOG, Eastern Cooperative Oncology Group; HBs, Hepatitis B surface; HCV, Hepatitis C virus; HIV, Human Immunodeficiency Virus; PS, Performance Status.

### Sample size justification

This single-center study will be conducted based on an exploratory, proof-of-concept protocol of a tumor-naïve, plasma-only ctDNA approach. The planned enrollment of 10 patients is chosen pragmatically to evaluate operational feasibility (accrual, predefined sampling adherence, centralized assay success, logistics and data completeness) and to generate preliminary clinical data (within-subject ctDNA-to-recurrence lead time and concordance with standard surveillance). Given the exploratory intent and the high analytical cost per sample, no formal power calculation or confirmatory hypothesis testing is planned. Findings inform effect-size assumptions, variability, and operational parameters to design a subsequent multicenter, statistically powered study.

### Study endpoints

#### Primary endpoint.

The primary endpoint of this study is the interval between the detection of ctDNA positivity and the clinical diagnosis of recurrence. This interval is calculated only for patients who exhibit ctDNA positivity and documented recurrence, allowing assessment of the lead time provided by ctDNA detection. ctDNA positivity is defined as the identification of at least one mutation in a cancer-related gene through plasma analysis. This endpoint highlights the potential of ctDNA as an early warning system, offering lead time for therapeutic interventions before clinical recurrence becomes evident.

#### Secondary endpoints.

The secondary endpoints are as follows: (1) Concordance of genetic mutations between pre- and postoperative plasma samples, highlighting tumor evolution; (2) DFS at 3 years; overall survival (OS) at 5 years in association with landmark ctDNA status; (3) presence or absence of ctDNA positivity at the time of recurrence; and (4) correlations between the ctDNA status and clinicopathological features, such as tumor grade and lymphovascular invasion. Additionally, as secondary endpoints, we evaluate the prognostic impact of ctDNA status on DFS and OS across the entire cohort using Kaplan–Meier survival curves, log-rank tests, and Cox proportional hazards models. Univariate and multivariate analyses are conducted, with results reported as hazard or odds ratios, as appropriate. DFS is defined as the time from curative hepatectomy to either the first documented recurrence or death from any cause. OS is defined as the time from curative hepatectomy to death from any cause.

#### Follow-up.

Patients will be followed for up to 5 years postoperatively in accordance with clinical guidelines. Plasma samples will be longitudinally collected at predefined intervals: preoperatively (within 4 weeks prior to surgery) and postoperatively at 4, 12, 24, 36, and 48 weeks. Routine clinical assessments, including CT imaging and tumor marker evaluations (e.g., carcinoembryonic antigen, carbohydrate antigen 19−9), will also be conducted. During the 1st year, CT imaging will be performed at 4, 12, 24, 36, and 48 weeks postoperatively. Beyond the 1st year, CT imaging will be conducted every 6 months for the remainder of the 5-year follow-up period. If clinical or radiological evidence of recurrence is detected, ctDNA sample collection will be discontinued at that point. Decisions regarding adjuvant chemotherapy are left to the discretion of the treating physician; however, ctDNA results will remain blinded to physicians to prevent bias in treatment decisions. ACT usage will be recorded and incorporated into exploratory analyses. Patients are monitored for recurrence and survival outcomes over the 5-year postoperative period.

#### Plasma-Safe-SeqS technology.

The ctDNA analyses in this study are performed using the Plasma-Safe-SeqS platform, a highly sensitive next-generation sequencing-based technology [10]. This platform is capable of detecting tumor-specific mutations in as few as four mutant molecules, with sensitivity dependent on DNA input. For 10,000 GE of DNA input, the platform achieves a mutant allele frequency detection limit as low as 0.04% (cutoff is 4MM, input quantity range is 1,000–20,000 GE). In this study, 4,100 GE of DNA input is used, corresponding to a mutant allele frequency sensitivity of 0.1%. This capability enables precise and dynamic recurrence monitoring. The platform utilizes a standardized 14-gene panel, including *AKT1, APC, BRAF, CTNNB1, ERBB3, FBXW7, KRAS, NRAS, PIK3CA, POLE, PPP2R1A, RNF43, SMAD4,* and *TP53*. This comprehensive panel covers key genetic mutations commonly associated with CRC and provides detailed molecular profiling. By focusing solely on plasma-derived ctDNA, this technology eliminates the dependency on tumor tissue, streamlining the diagnostic process and reducing logistical barriers. In this feasibility-oriented study, the gene panel and blood sampling time points are intentionally limited to a predefined set to streamline the clinical workflow while enabling meaningful analysis. This study does not include tissue-based genomic comparison with the primary tumor or metastatic site, as it aims to evaluate the performance of a tumor-naïve plasma-only assay that can be implemented even when tumor tissue is unavailable.

The ctDNA testing is conducted under a contractual agreement with Sysmex Corporation (Kobe, Japan), which performs the analyses using their proprietary Plasma-Safe-SeqS platform. All plasma samples are sent to Sysmex for centralized processing and analysis, ensuring consistency and quality control throughout the study. The typical turnaround time for the ctDNA assay using the Plasma-Safe-SeqS platform is expected to be approximately 10–14 business days from sample collection to result reporting.

### Statistical analysis

We implement an embedded comparator using standard surveillance under clinician blinding. This embedded comparator within individuals provides the frame necessary to evaluate ctDNA performance without a randomized control group. Analyses include the following: (i) lead time from first ctDNA positivity to clinical/radiologic recurrence; (ii) concordance describing which modality (ctDNA vs. standard surveillance) first identified recurrence; and (iii) landmark diagnostic performance (12 and 24 weeks) estimating sensitivity, specificity, PPV, and NPV for ctDNA relative to eventual recurrence status. Results are summarized descriptively and visualized with Swimmer’s plots. As the protocol is exploratory, no causal efficacy comparison is attempted; findings inform the design of a subsequent powered, large multicenter study.

Kaplan–Meier methods are used to estimate the 3-year DFS and 5-year OS rates, providing survival probabilities over time. Exploratory Cox regression using landmark analysis is conducted to evaluate associations between ctDNA status at predefined time points (e.g., 12 or 24 weeks) and clinical outcomes, such as recurrence risk and survival. Kaplan–Meier curves and log-rank tests will be used to compare 3-year DFS and 5-year OS by landmark ctDNA status. Owing to the limited number of patients, time-varying covariates are not used. A Swimmer’s plot will also be used to visualize individual patient timelines, including ctDNA positivity and recurrence events. Univariate and multivariate analyses will be conducted, and results will be reported using hazard ratios or odds ratios, as appropriate. Fisher’s exact test will be used to evaluate the concordance of genetic mutations between pre- and postoperative plasma samples, offering insights into tumor evolution. Statistical significance is set at *p* < 0.05. These analyses aim to validate the clinical utility of plasma-only ctDNA assays and guide the design of future larger-scale trials.

### Ethics approval and consent to participate

This single institute prospective observational study was approved by the Institutional Review Board of Osaka General Medical Center (approval number: 2024−052) (protocol ver1, November 6, 2024). All participants provide written informed consent in accordance with the principles of the Declaration of Helsinki.

### Data collection and management

Clinical data, including patient demographics, treatment details, and outcomes, will be collected in a secure, anonymized database. All ctDNA results will remain blinded to treating physicians throughout the study, preventing potential bias in clinical decision-making and ensuring the validity of the study outcomes. Plasma samples will be processed and analyzed at a centralized laboratory to ensure consistency, with stringent quality control measures implemented to maintain data integrity. These measures include duplicate analyses for key samples, standardized protocols for DNA extraction and sequencing, and regular calibration of laboratory equipment to ensure reproducibility and reliability.

## Discussion

The CASSIOPEIA study represents a significant advancement in the clinical application of ctDNA as a non-invasive biomarker for early recurrence detection in patients with resected CRC liver metastases. By exclusively utilizing plasma-derived ctDNA without requiring tumor tissue, this study simplifies the diagnostic process, addressing critical challenges associated with current ctDNA methodologies, such as the need for high-quality tumor DNA, extended turnaround times, and logistical issues. This approach enhances the applicability of ctDNA analysis for integration into routine clinical practice and expands its scalability. Moreover, this streamlined methodology could facilitate earlier detection of recurrence, enabling timely therapeutic interventions that potentially improve survival outcomes for patients.

To our knowledge, this study is the first to employ a 14-gene panel using Plasma-Safe-SeqS technology for ctDNA analysis in patients with CRC liver metastases following curative resection. The Plasma-Safe-SeqS platform provides a highly sensitive method for detecting tumor-derived genetic mutations. The platform can detect as few as four mutant molecules, with sensitivity varying by input DNA amount. At 10,000 GE of DNA input, the detection limit is 0.04%, whereas at 4,100 GE, as used in this study, the sensitivity is 0.1% [10]. This high sensitivity ensures precise monitoring of recurrence risk and offers a streamlined workflow that eliminates the dependency on tumor tissue.

Currently, ctDNA methodologies predominantly rely on tumor-informed assays, which require prior knowledge of tumor-specific mutations derived from tumor tissue [6,7,9]. Although tumor-informed approaches are highly sensitive, they introduce practical limitations. These include the necessity for high-quality tumor DNA, which may not always be available, especially in cases where patients have received treatment at multiple institutions or where tumor samples degrade over time. Additionally, the requirement for tumor DNA sequencing and personalized assay design extends the turnaround time, delaying actionable clinical decisions. These challenges restrict the application of tumor-informed assays in broader clinical settings.

In contrast, tumor-naïve approaches, such as the plasma-only methodology employed in this study, overcome these limitations by directly analyzing ctDNA from plasma. This approach accelerates diagnostic workflows, captures broader tumor heterogeneity, and eliminates logistical dependencies on tumor tissue [13]. These advantages make plasma-only methodologies a practical and scalable option for routine clinical use. However, a consensus on whether tumor-informed or tumor-naïve assays are superior remains elusive to date. Each method has distinct advantages and limitations, requiring further studies to determine their optimal use and how they can complement each other in clinical practice. Furthermore, broader operational considerations must be addressed to facilitate the real-world implementation of plasma-only ctDNA assays. These include evaluating cost-effectiveness, particularly in resource-limited settings, and ensuring accessibility across diverse healthcare systems. Regulatory approvals are also a critical step to enable widespread adoption, alongside efforts to standardize protocols for ctDNA testing. Additionally, integration into routine clinical workflows requires collaboration between healthcare providers, laboratories, and policymakers to establish guidelines for when and how ctDNA testing should be employed. Addressing these challenges is essential for translating the findings of this study into real-world applications and ensuring the scalability and sustainability of this approach.

This study focuses on homogenous patients with CRC liver-only metastases, a population at high risk for recurrence after curative resection. Liver metastases are the most common site of distant spread in CRC and significantly contribute to recurrence and mortality. Despite advancements in surgical and systemic therapies, recurrence rates remain alarmingly high, underscoring the need for innovative surveillance strategies that extend beyond traditional imaging and tumor markers [2]. Traditional imaging methods, such as CT scans, often lack the sensitivity to detect micrometastatic disease, whereas tumor markers such as carcinoembryonic antigen are nonspecific and may lead to delayed detection of recurrence. These limitations highlight the potential of ctDNA-based monitoring to provide earlier and more precise detection of recurrence, addressing a critical gap in the management of high-risk populations.

In addition to recurrence detection, ctDNA offers significant potential to guide the use of adjuvant chemotherapy in the postoperative setting. Identifying ctDNA-positive patients postoperatively enables the targeted administration of adjuvant therapy, ensuring high-risk patients benefit from treatment while sparing ctDNA-negative patients from unnecessary toxicity [9]. Currently, clinical trials investigating adjuvant chemotherapy in patients with CRC with resected liver metastases have yielded inconclusive results, with limited benefit observed in certain studies [3,4]. Consequently, routine administration of adjuvant chemotherapy for all patients cannot be uniformly recommended. By integrating ctDNA status into decision-making, this study aims to refine postoperative treatment strategies and enable personalized care.

One of the primary strengths of this study is its focus on dynamic ctDNA monitoring at predefined intervals. This design provides a detailed understanding of ctDNA kinetics and their temporal patterns, which could help identify critical windows for intervention. The inclusion of pre- and postoperative plasma samples facilitates the evaluation of concordance in genetic mutations, offering insights into tumor evolution, mechanisms of resistance, and the potential impact of surgical intervention on ctDNA dynamics. Additionally, by assessing the DFS and OS rates in association with landmark ctDNA status, the study explores the broader implications of ctDNA status on long-term clinical outcomes, providing a foundation for future intervention strategies and treatment optimization.

Despite its strengths, this study has some limitations. As a single-center observational trial, the findings may not be immediately generalizable to broader or more diverse clinical settings. Multicenter trials with diverse patient populations could help validate the broader applicability of plasma-only ctDNA methodologies and account for variations in clinical practices across institutions. Additionally, the relatively small sample size restricts the ability to perform detailed subgroup analyses or achieve statistically significant conclusions. Nevertheless, the primary aim of this study is to provide essential proof-of-concept data, which serve as a foundation for designing larger multicenter trials to validate ctDNA-based surveillance and treatment strategies. The Plasma-Safe-SeqS platform offers high sensitivity, detecting mutations with a mutant allele frequency as low as 0.04% for 10,000 GE of DNA input (cutoff is 4MM, input quantity range is 1,000 to 20,000 GE). However, its sensitivity is somewhat lower than that of tumor-informed methods such as Signatera, which has a limit of detection of ~0.01% and sensitivity of >95% [7–9,14]. In addition, its performance has not been directly benchmarked against other ctDNA detection technologies, such as Guardant Reveal or tumor-informed approaches. Furthermore, the reliance of the platform on a standardized 14-gene panel may limit its capability to detect rare mutations in highly heterogeneous tumor profiles. Future comparative studies between plasma-only and tumor-informed ctDNA methodologies are crucial to establish their respective advantages and optimize their clinical applications. Additionally, the integration of advanced approaches such as whole-exome sequencing or whole-genome sequencing could provide a more comprehensive understanding of tumor heterogeneity and uncover novel biomarkers for recurrence and treatment response [15]. These techniques may play a pivotal role in future research, enabling the refinement of ctDNA analysis and expanding its clinical utility.

In conclusion, the CASSIOPEIA study represents a significant step forward in validating a plasma-only ctDNA approach for postoperative surveillance and therapeutic decision-making in patients with CRC liver metastases. By addressing the challenges associated with tumor-informed assays, this study demonstrates the clinical utility of integrating ctDNA analysis into routine workflows. Beyond recurrence detection, ctDNA analysis holds promise for transforming postoperative management by identifying patients who could benefit from adjuvant chemotherapy. These findings are expected to advance personalized medicine and improve outcomes for patients with CRC liver metastases. Moreover, this study establishes a foundation for incorporating plasma-only ctDNA analysis into clinical guidelines, paving the way for its standardized use in postoperative surveillance.

## Supporting information

S1 File CASSIOPEIA protocol in English translation. (DOCX)

S2 File CASSIOPEIA protocol in Japanese. (DOCX)

S3 File SPIRIT-Outcomes-2022-Checklist-with-SPIRIT-2013. (PDF)

S4 File SPIRIT figure. (PDF)

## Abbreviations

CRC: Colorectal Cancer;
ctDNA: Circulating Tumor DNA
DFS: Disease-Free Survival
OS: Overall Survival
GE: Genome Equivalents
ACT: Adjuvant Chemotherapy
