# Supplementary material for: Clinical utility of circulating tumor DNA for early detection of recurrence after curative hepatectomy in patients with colorectal cancer with liver metastases: A prospective observational study protocol (CASSIOPEIA)
Source: PLoS One. 2025 Nov 20;20(11):e0335591. doi: 10.1371/journal.pone.0335591 (PMC12633885; doi:10.1371/journal.pone.0335591)
Supplement: S1 File — (DOCX) [file pone.0335591.s001.docx]

**Blood circulating tumor DNA after radical resection of liver metastases of radically resectable colorectal cancer.**

**The purpose of this study was to examine whether measuring the early detection of recurrence is useful for the early detection of recurrence.**

**Single-center prospective observational study**

**ClinicAl utility of circulating tumor DNA to detect early tumor recurrence**

**after Surgery in patients with radically reSectable lIver metastases from cOlorectal cancer: a single institute ProspEctive observatIonAl study**

**CASSIOPEIA**

**Research Implementation Plan**

Date Prepared　　October 15, 2024 Draft Plan Version 1 prepared.

**0.0 Overview**

**0.1. overview of this study**

In this study, we will examine whether the measurement of cancer-related genes with genetic mutations is useful for early detection of recurrence in patients with colorectal cancer who have only liver metastases that can be radically resected, by performing the same gene panel test using blood samples from before and after radical resection of the liver metastases. Furthermore, we aim to explore the usefulness of this measurement system in Japanese clinical practice and the significance of planning a large-scale clinical trial with a view to insurance reimbursement.

**schema
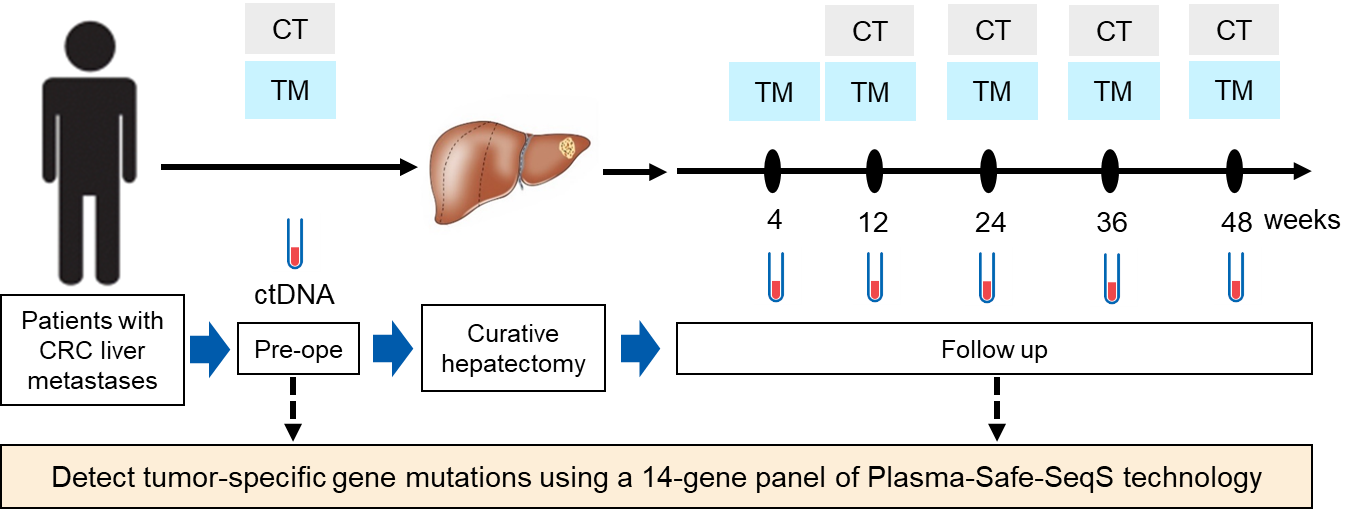
**

**0.2. purpose**

The purpose of this study is to investigate whether measuring cancer-related genes with genetic mutations is useful for early detection of recurrence in patients with colorectal cancer who have only liver metastases that can be radically resected, by performing the same gene panel test using blood samples from pre-/postoperative radical resections for liver metastases.

**0.3. research subjects**

**0.3.1. selection criteria**

Cases that meet all of the following criteria will be considered

(1) Histopathologically diagnosed as adenocarcinoma of the colon.

(ii) The primary site is diagnosed as colon (cecum, colon, sigmoid rectum) or rectum, and the primary site has been resected (excluding appendiceal and anal canal cancer).

(iii) The patient has no distant metastases other than liver metastases and is scheduled for initial radical resection for colorectal cancer liver metastases.

　　 (Conversion Therapy" cases in which radical resection of liver metastases is possible after systemic drug therapy are also eligible.)

(iv) The patient is 20 years of age or older on the date of consent.

Eastern Cooperative Oncology Group (ECOG) Performance Status (PS) 0 or 1.

(6) Written consent to participate in this study has been obtained from the individual.

**0.3.2 Exclusion Criteria**

Cases meeting any of the following criteria shall be excluded

(1) Active overlapping cancer is present at the time of liver resection.

However, patients with a recurrence-free period of at least 5 years, or with basal or spinous cell carcinoma of the skin deemed curable by local treatment, superficial bladder cancer, cervical cancer, non-invasive ductal carcinoma, carcinoma in that can be treated endoscopically (intraepithelial carcinoma) or lesions equivalent to intramucosal carcinoma, and nonmetastatic prostate cancer that does not require systemic treatment may be enrolled. Patients with metastatic prostate cancer that does not require systemic treatment may be enrolled.

Pregnant or lactating woman.

(iii) The physician in charge judges that the patient is inappropriate as a research subject of this study.

**0.4. endpoints**

**0.4.1. primary endpoint**

Assess the interval between the time of diagnosis of recurrence and the time of positive blood circulating tumor DNA (ctDNA).

**0.5. target number of cases and duration of study**

**0.5.1. target number of patients:** 10 colorectal cancer patients with liver metastasis only who are scheduled for radical resection at our institution

**0.5.2. Registration period:** December 1, 2024 - December 1, 2025

**0.5.3. tracking period:** 12/1/2024 - 12/1/2026

**0.5.4. entire study period:** December 1, 2024 - December 1, 2027

**0.6. research methods**

**0.6.1. type and design of study**

In this study, samples will be collected and stored in a prospective case series, and samples will be measured and analyzed together at the end of the follow-up period.

**0.6.2. Method of Observation**

DNA will be extracted from blood samples collected pre-/post-operatively for radical resection of liver metastases, and identical gene panel tests using the Plasma-Safe-Seq (PSS) method will be performed to measure cancer-related gene mutations in individual cases. All of these measurements will be performed together after the end of the follow-up period.

**0.7. research implementation system**

This study will be conducted under the following structure

Research Assistants]

0 Inoue, Gastroenterological Surgery (Principal Investigator)

Yujiro Nishizawa Gastroenterological Surgery

　　　　Yoshiyu Morimoto Gastroenterological Surgery

　　　　Yuki Ohsato Gastroenterological Surgery

　　　　Masahiro Hashimoto Gastroenterological Surgery

[Contact].

Osaka Acute & Comprehensive Medical Center

digestive surgery

Location: 3-1 Bandaihigashi, Sumiyoshi-ku, Osaka, 558-8558, Japan− 56

Phone: 06-6692-1201

E-mail: inoue_akira@gh.opho.jp

**0.8. ethical matters**

The research will be conducted in accordance with the guidelines established by the government ("Ethical Guidelines for Life Science and Medical Research Involving Human Subjects").

**order of precedence**

0.0 Overview 1

Background of the Study 4

Purpose of the study 6

3. target number of cases and study period 6

4. research subjects 6.

5. research methods 6

6. Endpoints 11.

7. completion of research 11

8. discontinuance criteria 11

9. anticipated benefits and disadvantages (side effects) associated with the study 12.

10. change, discontinuance, suspension or termination of research 12

11. method of obtaining consent 12.

12. handling of personal information 13.

13. the need for and availability of genetic counseling in the conduct of research 13

14. statistical matters 13

15. storage and disposal of samples and information (including materials pertaining to information used in research) Methods of storage and disposal of samples and information (including materials pertaining to information used in research) 14. Methods of storage and disposal of samples and information (including materials related to information used in research)

16. contents and method of reporting to the head of the research organization 14.

17. research expenses and conflicts of interest 15

18. details of financial burden or gratuities to research subjects, etc. 15.

19. compensation for damage to health 15

20. attribution of research results and publication of research results 15

21. handling of results, etc. obtained in research 15.

22. research implementation system 15.

23. list of references and bibliography 16

**Background of the study**

**1.1. epidemiology of colorectal cancer**

The morbidity and mortality of colorectal cancer in Japan remain high, and the development of methods for its prevention, early diagnosis, and treatment is an extremely important issue. The annual number of deaths from colorectal cancer in 2018 was 27,098 in men and 23,560 in women, ranking third in men and first in women by carcinoma type.(1) The 5-year survival rates for colorectal cancer patients who underwent radical resection for colorectal cancer are Stage I: 91.6%, Stage II: 84.8%, Stage IIIa: 77.7%, Stage IIIb: 60.0%, and Stage IV: 18.8%.(2)

**1.2. standard treatment for colorectal cancer**

Radical surgical resection is performed for Stage I to III colorectal cancer. The standard postoperative treatment for Stage I/II patients without pathologic lymph node metastasis is follow-up, while postoperative adjuvant chemotherapy is recommended for Stage III patients with pathologic lymph node metastasis. For Stage IV patients with distant metastases and recurrent cases, surgical resection of metastases is the standard of care when radical surgical resection is possible, with perioperative chemotherapy as an option depending on the risk. On the other hand, if radical surgical resection is difficult, systemic chemotherapy is used. In addition, standard follow-up for colon and rectal cancer after radical surgical treatment is recommended by the Guidelines for the Treatment of Colorectal Cancer (Colorectal Cancer Study Group, 2024 edition), which include a history and examination, CT scan, tumor markers, and endoscopy on a regular basis.(2)

**1.3. standard treatment for colorectal cancer with liver metastasis and clinical issues**

Liver metastases are the most frequent distant metastases of colorectal cancer. The standard treatment for liver metastases that can be curatively resected is surgical resection. However, the recurrence rate after resection of distant metastases is as high as 50% to 70%, and the use of postoperative adjuvant chemotherapy has been discussed to improve treatment outcomes.(3, 4) However, some patients recur after resection of liver metastases and others do not, and it is not clear which patients should be aggressively treated with postoperative adjuvant chemotherapy. Recent studies have shown that patients who recur have minimal residual disease (MRD), and although there is a study(5, 6) that reported that detecting MRD after resection of liver metastases was useful in assessing the risk of recurrence, this has not yet been established in actual clinical practice. Therefore, only a small percentage of patients currently benefit from adjuvant chemotherapy, and nearly half of patients do not recur without chemotherapy. Such patients receive unnecessary chemotherapy with side effects, which may significantly impair their quality of life. Thus, the clinical challenge is to tailor postoperative adjuvant chemotherapy to the individual patient's risk of recurrence.

Recently, the results of the JCOG0603 trial were published in a paper from Japan at(7) . This randomized trial compared postoperative adjuvant chemotherapy (mFOLFOX6) with surgery alone in patients after curative resection of liver metastases from colorectal cancer. Results showed that the primary endpoint, disease-free survival (DFS), was significantly prolonged with mFOLFOX6. However, no difference in overall survival (OS) was demonstrated, and the interpretation in clinical practice is still inconclusive. In summary, colorectal cancer treatment guidelines and previous reports currently weakly recommend postoperative adjuvant chemotherapy because the prevention of recurrence itself may benefit the patient in terms of avoiding another liver resection, a highly invasive treatment.(2, 3)

**1.4. association between colorectal cancer and genetic abnormalities**

Genetic abnormalities have been shown to play a major role in the development and progression of colorectal cancer, and in The Cancer Genome Atlas Network (TCGA) project study, a whole genome analysis was conducted using clinical samples from 276 colorectal cancer cases.(8) The results revealed that colorectal cancers are divided into two types: Hypermutated-type with many gene variants (mutations) and Nonhypermutated-type without many gene variants (mutations). In the former type, ACVR2A (63%), APC (51%), TGFBR2 (51%), and BRAF (46%) were detected as frequently aberrant genes, while APC (81%), TP53 (60%), KRAS (43%), and TTN (31%) were found in the latter type. The importance of genes involved in the WNT signaling pathway and TGF-β signaling pathway, which activate MYC, as potential therapeutic targets for colorectal cancer was again revealed. In addition, genetic abnormalities such as IGF2, IGFR, ERBB2, ERBB3, MEK, AKT, and MTOR were also observed, and it is expected that therapeutic agents targeting these driver alterations will be developed.

**1.5. blood circulating tumor DNA and liquid biopsy**

Recent research has revealed the presence of circulating tumor-derived DNA (circulating tumor DNA, ctDNA) that leaks from tumor tissue into the bloodstream. Liquid biopsy is a new technique for detecting ctDNA, mainly from blood, for cancer diagnosis , and is currently undergoing rapid clinical development. The advantage of liquid biopsy is that it is simpler and less invasive than conventional methods using tumor tissue because it can be performed only by drawing blood. Furthermore, it has many advantages, including the ability to test tumor tissue even when it is difficult to collect, the possibility of repeated collection, the ability to identify changes in cancer-related genetic abnormalities, the ability to analyze in a shorter time than tissue tests, and the ability to capture the overall picture of genetic abnormalities in tumors.(9)

Clinical applications of liquid biopsy in colorectal cancer include: 1) prognostic prediction/early response to chemotherapy

determination, (2) prediction of efficacy of molecular-targeted drugs/detection of acquired resistance mutations, (3) detection of MRD after curative surgical resection and evaluation of recurrence risk, and (4) profiling of cancer-related genetic abnormalities and selection of appropriate molecular-targeted drugs. Currently, research and development of genetic analysis using Next Generation Sequencing (NGS) for these purposes is underway.

A high recurrence rate has been reported in patients with ctDNA detected by liquid biopsy for colorectal cancer after radical resection(10) , and clinical trials are currently underway to select the content of postoperative adjuvant chemotherapy using liquid biopsy. In addition, patients with RAS mutations in advanced or recurrent colorectal cancer should be confirmed to be RAS wild-type prior to treatment, as it is likely that they will not benefit (prolong life or shrink tumor size) from anti-EGFR antibody therapy. The OncoBEAM^TM^ RAS CRC Kit, which evaluates the presence of RAS gene mutations from ctDNA in blood, is an in vitro diagnostic product using the BEAMing method developed by Sysmex, and was covered by insurance in Japan on August 1, 2020.

Thus, analysis of cancer gene mutations in liquid biopsy specimens using NGS and other methods is expected to enable more precise and rapid selection of drugs for ultra-early detection and treatment of cancer.(11) In Japan, a nationwide multicenter clinical trial initiative (SCRUM-Japan) led by the National Cancer Center Hospital East is being vigorously conducted ahead of the rest of the world, and a medical system for conducting such tests is being established in Japan.

**1.6. about Plasma-Safe-Seq technology and** **gene panel testing**

Plasma-Safe-Seq (hereinafter referred to as "PSS") technology was developed by medical device manufacturer Sysmex Corporation (Kobe, Hyogo, Japan) and is a new testing system to detect ctDNA in plasma. By using the DNA molecular barcode method, NGS reading errors can be prevented and The sensitivity of detection by NGS is improved more than 10-fold compared to conventional methods, enabling detection of mutant allele frequencies (MAF) of up to 0.05%. Clinical development using this technology is underway, and it has been reported that the presence or absence of ctDNA detection after radical resection predicts the risk of recurrence with high accuracy in 230 stage II colorectal cancer patients.(10) In addition, it has been reported that the presence or absence of ctDNA detection after radical resection and postoperative adjuvant chemotherapy was useful in assessing the risk of recurrence in locally advanced rectal cancer and stage III colon cancer.(12, 13)

A gene panel test using the PSS method is a test method that performs Target Sequence (target sequence analysis) on 14 genes that are frequently mutated in colorectal cancer.(10)

In our validation study conducted prior to this study, we performed a genetic panel test using the PSS method on four tissue samples from liver metastases (FFPE) to measure baseline genetic mutations and were able to actually identify cancer-related genes with genetic mutations in all four cases. In other words, APC gene mutations were found in 3 of the 4 cases. In addition, TP53 mutations were found in two of the four cases, and PIK3CA, NRAS, and SMAD4 mutations were found in one of each of the four cases.

The above results indicate that this technology can be used to identify genetic mutations with a high degree of accuracy, and the feasibility of this research is extremely high.

**1.7. significance of this study**

In this study, we will perform the same gene panel test on colorectal cancer patients with only liver metastases that can be radically resected, using pre- and postoperative blood specimens, to explore whether measuring cancer-related gene mutations is useful for the early diagnosis of recurrence after radical resection for liver metastases. If the usefulness of this testing system is confirmed, a clinical trial will be designed to apply for approval to the PMDA. In the future, clinical trials can be designed to evaluate selective postoperative adjuvant therapy according to the risk of recurrence in individual patients. As a further development, real-time profiling of cancer-related genetic abnormalities in blood samples is expected to enable the selection of appropriate molecular-targeted therapies for individual patients. In summary, we believe that this study is extremely significant because the development of new diagnostic and therapeutic approaches throughout the research will benefit colorectal cancer patients with liver metastases.

**Purpose of the study**

The purpose of this study is to investigate whether measuring cancer-related genes with genetic mutations is useful for early detection of recurrence in patients with colorectal cancer who have only liver metastases that can be radically resected, by performing the same gene panel test using blood samples from pre-/postoperative radical resections for liver metastases.

**3. target number of cases and study period**

**3.1. target number of patients:** 10 patients with colorectal cancer with liver metastases scheduled for curative resection at this facility

**Rationale:** Calculated based on the annual number of cases at the facility.

**3.2. registration period: December 1, 2024 - December 1, 2025**

**3.3. tracking period: december 1, 2024 - december 1, 2026**

**3.4. entire study period: December 1, 2024 - December 1, 2027**

**4. Research Subjects**

**4.1. selection criteria**

Cases that meet all of the following criteria will be considered

(1) Histopathologically diagnosed as adenocarcinoma of the colon.

(ii) The primary site is diagnosed as colon (cecum, colon, sigmoid rectum) or rectum, and the primary site has been resected (excluding appendiceal and anal canal cancer).

(iii) The patient has no distant metastases other than liver metastases and is scheduled for initial radical resection for colorectal cancer liver metastases.

　　 (Conversion Therapy" cases in which radical resection of liver metastases is possible after systemic drug therapy are also eligible.)

(iv) The patient is 20 years of age or older on the date of consent.

Eastern Cooperative Oncology Group (ECOG) Performance Status (PS) 0 or 1.

(6) Written consent to participate in this study has been obtained from the individual.

**4.2 Exclusion Criteria**

Cases meeting any of the following criteria shall be excluded

(1) Active overlapping cancer is present at the time of liver resection.

However, patients with a recurrence-free period of at least 5 years, or with basal or spinous cell carcinoma of the skin deemed curable by local treatment, superficial bladder cancer, cervical cancer, non-invasive ductal carcinoma, carcinoma in that can be treated endoscopically (intraepithelial carcinoma) or lesions equivalent to intramucosal carcinoma, and nonmetastatic prostate cancer that does not require systemic treatment may be enrolled. Patients with metastatic prostate cancer that does not require systemic treatment may be enrolled.

Pregnant or lactating woman.

(iii) The physician in charge judges that the patient is inappropriate as a research subject of this study.

**5. research methods**

**5.1. overview of this study**

The schematic of this study is shown in the figure below. The subjects of this study are patients with colorectal cancer who have only liver metastases that can be curatively resected. Preoperative/postoperative blood samples from patients undergoing radical resection for liver metastases will be collected and tested for the same gene panel using the PSS method to determine cancer-related gene mutations. Samples will be collected and stored prospectively, and samples will be compiled, measured and analyzed retrospectively after the end of the follow-up period. The purpose of this study is to determine whether the same gene panel test using preoperative/postoperative blood samples from patients undergoing radical resection for liver metastases and measuring cancer-associated gene mutations is useful for early detection of recurrence.

**schema**

**
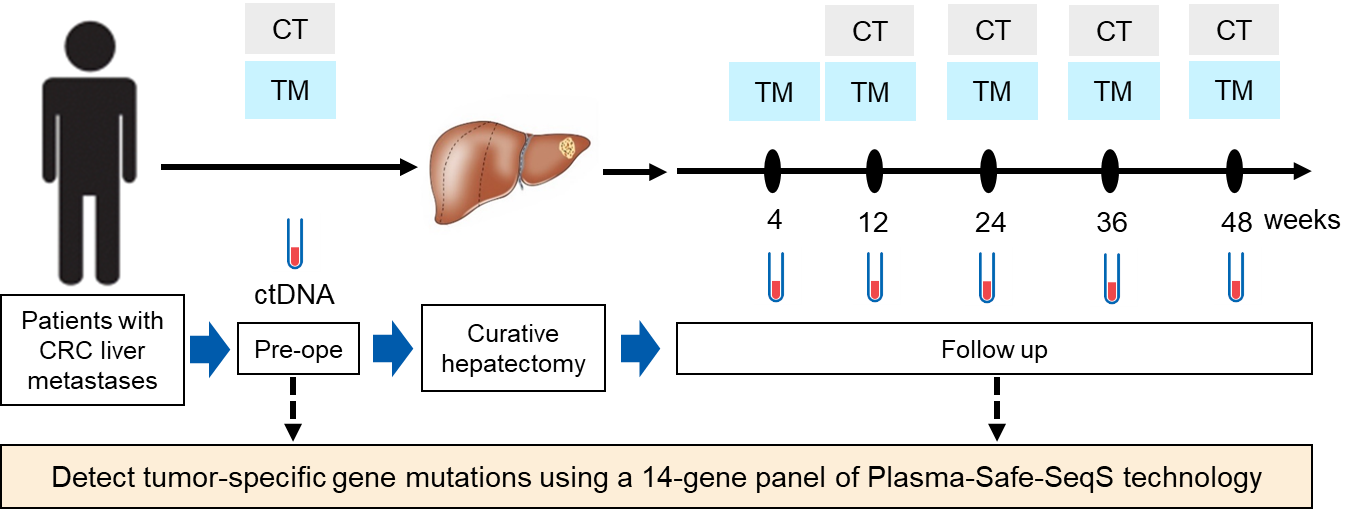
**

**5.2. type and design of study**

In this study, samples will be collected and stored in a prospective case series, and samples will be measured and analyzed together at the end of the follow-up period.

**5.3. methods of observation**

DNA will be extracted from blood samples collected pre-/post-operatively for radical resection of liver metastases, and identical gene panel tests using the Plasma-Safe-Seq (PSS) method will be performed to measure cancer-related gene mutations in individual cases. All these measurements will be performed together after the end of the follow-up period.

**5.4. how to enroll research subjects**

　　 The Principal Investigator and the Principal Investigator will confirm the eligibility of research subjects whose consent has been obtained in writing, and enroll those who are deemed eligible into the study as research subjects.

**5.5. information on drugs, medical devices, treatments, etc., and methods of use of the drugs, medical devices, and treatments that are the subject of the research**

In the validation study that preceded this study, tissue samples from liver metastases (FFPE) were analyzed for genes using the Sysmex PSS gene panel test, a test that performs Target Sequence (TSE) analysis on 14 genes that are frequently mutated in colorectal cancer. Target Sequence Analysis) for 14 genes with high mutation frequency in colorectal cancer.(10) In a validation study we conducted prior to this study, the same gene panel test was performed on tumor tissue (liver metastases) and blood samples from individual patients to measure cancer-related gene mutations.

In our validation study prior to this study, we performed a genetic panel test using the PSS method on four tissue samples from liver metastases (FFPE) to measure baseline gene mutations and were able to actually identify cancer-associated gene mutations in all four cases with genetic variants (Table 1). In other words, APC gene mutations were found in 3 of the 4 cases. In addition, TP53 gene mutation was found in 2 out of 4 cases, and PIK3CA gene mutation, NRAS gene mutation and SMAD 4 gene mutation were found in 1 out of 4 cases each.

(Table 1)

| **Patient Information** | **Gene Name \| Sequence Variation \| Mutation Frequency** |
| --- | --- |
| 67 years old Male  Transverse colon cancer, iatrogenic liver metastasis | APC \| c.4037C>G \| 13.699% (c.847C>T \| 23.37%)  APC \| c.847C>T \| 23.37  TP53 \| c.742C>T \| 30.832% (c.742C>T \| 30.832%) |
| 70 years old Male  Rectal cancer Concurrent liver metastasis | APC \| c.4057G>T \| 5.515% (*)  APC \| c.637C>T \| 5.283% (APC) |
| 78 years old male  Rectal cancer Concurrent liver metastasis | APC \| c.4135G>T \| 16.292% (c.904C>T \| 37.215%)  APC \| c.904C>T \| 37.215% (APC)  PIK3CA \| c.1633G>A \| 17.922% APC |
| 45 years old, female.  Sigmoid colon cancer, iatrogenic liver metastasis | NRAS \| c.182A>T \| 42.024% (c.182A>T)  SMAD4 \| c.1082G>A \| 68.634% (c.1082G>A)  TP53 \| c.422G>A \| 70.527% (c.422G>A) |

**5.6. survey items and clinical examination**

**5.6.1. background of study subjects**

Collect the initials, facility case number, gender, and birth date or age (at time of enrollment) of the research subject.

In addition, the following items and others will be investigated for the underlying disease.

1) General condition

Performance status (ECOG)

Complications, history, medications, smoking history

2) Information on primary organs

Primary site

Date of initial diagnosis

Pathological histology

Clinical staging in the UICC 8th edition after resection of primary tumor

3) Diagnostic imaging taken in daily practice

CT scan

MRI scan

PET-CT Examination

Gastrointestinal endoscopy

4) Information on tumor markers and genetic mutations tested in routine medical care

Tumor markers (CEA, CA19-9, p53)

Gene mutations (RAS, BRAF, MSI, MMR, HER2, TMB, NTRK, RET, and other gene mutation information obtained from gene panel tests)

5) Previous treatment history

Chemoradiotherapy or chemotherapy prior to curative resection

Information on initial surgery (primary tumor resection)

**5.6.2 Post-registration clinical course**

The following items and others will be investigated after enrollment in this study

1) Pathological diagnosis after resection of liver metastases

Pathological classification in the UICC 8th edition and the 9th edition of the colorectal cancer code of conduct at the time of surgery

Pathological findings (pathological histology, wall depth, invasive growth pattern, lymphatic invasion, venous invasion, coelution, proximal and distal sections, presence or absence of exposed cancer invasion in deep sections, multiple cancers, lymph node metastasis, distant organ metastasis, residual cancer, degree of cure, etc.)

2) Diagnostic imaging taken in daily practice

CT scan

1. Starting from the date of surgery, every 12 weeks (±2 weeks allowed, same day of the week allowed) until 48 weeks.
2. If enrolled in a study associated with this observational study, imaging evaluations will be performed at the intervals specified for each study.

MRI scan

PET-CT Examination

Gastrointestinal endoscopy

3) Tumor markers tested in daily practice

CEA, CA19-9

1. Starting from the date of surgery, the procedure should be performed every 12 weeks (±2 weeks allowed, same day of the week is acceptable) until 4 weeks and 48 weeks postoperatively.
2. If enrolled in a study associated with this observational study, blood tests will be performed at the intervals specified for each study

4) Clinical course

Operation

Perioperative complications (Clavien Dindo Grade 2 or higher)

If perioperative treatment was received, regimen, treatment start date, and last date of administration

Date of recurrence

Date of Last Survival Confirmation

Treatment after recurrence

**5.6.3. tissue samples**

In this study, it is envisioned that tissue samples will be analyzed as needed. FFPE (formalin-fixed paraffin-embedded) specimens prepared from tumor tissue (liver metastases) surgically resected for liver metastases of colorectal cancer at our institution will be used. The amount of specimen required for analysis (25 mm^2^, 10 µm thick, 8 slides, tumor content >20%) should be sent to the laboratory. If necessary, this tumor tissue specimen will be used for genetic testing. In addition, the association with outcomes and other clinicopathologic and molecular biological features will be evaluated. All of these measurements and analyses will be performed together at the end of the follow-up period.

The handling of samples is in accordance with the Regulations for Handling Histopathology Specimens for Genome Research and Clinical Use(14) .

**5.6.4. blood specimens**

In this study, 20 ml of blood will be collected for research purposes before surgery (within 4 weeks before surgery, same day of the week allowed), 4 weeks after surgery (±1 week allowed, same day of the week allowed), and every 12 weeks after surgery (±2 weeks allowed, same day of the week allowed) until 48 weeks after surgery, respectively. Plasma should be centrifuged and stored in an ultra-low temperature freezer (-70°C or lower) according to the protocol at this institution. (Refer to the CASSIOPEIA specimen collection manual for details.) Once a relapse is diagnosed, the blood sample should be collected and the collection of blood samples for further studies should be terminated. On the other hand, if the relapse occurs at a time beyond 48 weeks postoperatively, blood specimens should be collected at that time. In cases where blood samples cannot be collected during the prescribed collection period, the responsible physician may decide to collect blood samples for research purposes outside of the prescribed collection period. Blood samples for CEA and CA19-9 and imaging tests will be collected during the observation period specified in this study. In addition to the above, the study blood samples will not be collected after 4 weeks postoperatively, since patients who underwent R2 resection will be omitted from the final analysis. Furthermore, if there is a history of blood transfusion within 2 weeks of the blood collection for the study, the date of the test will be changed or the blood collection will not be conducted at the relevant time if it is outside the allowable date, as it will affect the test results.

If, after enrollment in this study, the patient is enrolled in a study related to this study, testing will also be performed on the dates stipulated in the respective study. Blood samples will be sent from our institution to Sysmex Corporation (Kobe, Japan). Plasma will be subjected to ctDNA analysis using the PSS gene panel test. All of these measurements and analyses will be performed together after the end of the follow-up period.

The handling of samples is in accordance with the Guidelines for the Collection of Genomic Samples and Handling of Genomic Data(15) .

**5.7. test items and test schedule for this study**

|  | Before registration | date of surgery | Up to 48 weeks postoperatively | | | | |
| --- | --- | --- | --- | --- | --- | --- | --- |
|  |  |  | (~1 year) | | | | |
| Postoperative period |  |  | 4 weeks | 12 weeks | 24 weeks | 36 weeks | 48 weeks |
| tolerance level |  |  | ±1 week | ±2 weeks | ±2 weeks | ±2 weeks | ±2 weeks |
| Obtaining Consent | ○ |  |  |  |  |  |  |
| Selection/Exclusion Criteria | ○ |  |  |  |  |  |  |
| ECOG PS | ○ |  |  |  |  |  |  |
| lesion evaluation | ○ |  |  |  |  |  |  |
| Surgical and pathological findings |  |  | ○++ |  |  |  |  |
| previous medical treatment | ○ |  |  |  |  |  |  |
| CEA, CA19-9 | ○ |  | ● | ●+ | ●+ | ●+ | ●+ |
| return to origin |  |  | ▲ | ▲ | ▲ | ▲ | ▲ |
| CT of thoracoabdominal pelvic region | ○ |  |  | ●+ | ●+ | ●+ | ●+ |
| Total colonoscopy ^1)^ | ○ |  |  |  |  |  | ＊ |
| tumor tissue |  | ○+ |  |  |  |  |  |
| Blood collection for ctDNA |  | ◎ | ● | ●+ | ●+ | ●+ | ●+ |

Blood tests such as CEA and CA19-9, thoracoabdominal pelvic CT scan, and total colonoscopy will be collected from routine medical examinations.

1) *: If the entire colon cannot be observed preoperatively or postoperatively, it should be performed within 1 year after registration. Thereafter, the colonoscopy should be performed every year if neoplastic lesions are present, or every 3-5 years if not present.

0: within 4 weeks prior to registration (including the same day of the week)

0+: Tumor tissue will be sent to the laboratory from our facility in the form of FFPE thin slides.

0++: Surgical and pathology results will be kept at our facility as soon as the results are known.

◎ Within 4 weeks prior to surgery (including the same day of the week), but if preoperative treatment has been performed, from the end of preoperative treatment until prior to surgery.

●: Allow the specified date ± 1 week (including the same day of the week)

●+: Allow ±2 weeks (including the same day of the week) on the specified date (once a diagnosis of "relapse" as defined in 6.3.1. is made, subsequent blood samples for ctDNA will be terminated).

▲: Collect during this period

**5.8. actions to be taken after completion of the study**

After the completion of this study, the results will be analyzed and reviewed, and preparations will be made immediately for the next study. The results of this study will not affect the diagnosis or treatment plan of the patients.

**5.9. use of samples and information of other institutions, etc.**

nashi (Pyrus pyrifolia, esp. var. culta)

**Endpoints**

**6.1 Primary endpoint**

The interval between the time of diagnosis of relapse and the time of positive blood circulating tumor DNA (ctDNA) is evaluated. ctDNA positivity is defined as the identification of one or more cancer-related genes with genetic mutations in a blood test.

**6.2 Secondary endpoint**

The concordance rate of cancer-associated gene mutations in pre- and postoperative blood samples and their gene profiling will be evaluated. In addition, histopathological evaluation in tissue samples of liver metastases will be performed, and if necessary, cancer-related genes in tissue samples of liver metastases will also be analyzed. In addition, clinical outcomes such as Disease-free Survival (DFS), Overall Survival (OS), and ctDNA positivity at recurrence will be defined, and correlations among these outcomes and associations with other clinicopathological and molecular biological features will be explored and analyzed. The study will be conducted in an exploratory manner to analyze the correlation among these outcomes and the association with other clinicopathological and molecular biological characteristics.

**6.3. endpoint definition**

**6.3.1 Disease free survival (DFS)**

・ The period beginning on the date of surgery and ending on the earlier of the date of diagnosis of recurrence or the date of death from any cause. Recurrence" shall be determined on the basis of diagnostic imaging, and the date of the examination on which the imaging test was performed shall be the recurrence period. A period of worsening of the disease or elevation of tumor markers alone, not based on diagnostic imaging, shall not be considered a recurrence, and the date of examination when the recurrence is confirmed by diagnostic imaging shall be the diagnostic time of recurrence.

For imaging diagnosis of recurrence, the event is defined as the "date of examination" of the imaging test that yielded a "confirmed diagnosis", not the date of examination of the "imaging suspicion".

In the case of a surviving patient who has not been judged to have relapsed, the last date of confirmation of survival is the date of termination (confirmation of survival by telephone contact is also acceptable, but should be documented in the medical record). (Survival confirmation by phone call is also acceptable, but the fact that survival confirmation was made should be recorded in the medical record.)

When the definitive diagnosis of recurrence is made by biopsy pathology, if the diagnosis of recurrence cannot be made on imaging but is made by biopsy pathology, the event is considered to be the date of biopsy.

The occurrence of a secondary cancer (including iatrogenic multiple cancers and iatrogenic multiple cancers) is neither an event nor a censoring, but is considered DFS until another event is observed.

If the pathological findings show that the cancer remnant degree is R2, it is not included in the main analysis (see "14.3. Analysis Methods").

**6.3.2 Overall survival (OS)**

The period beginning on the date of surgery and ending on the date of death from any cause.

In surviving cases, the last date of confirmation of survival is the date of termination (telephone confirmation of survival is also acceptable. (Survival confirmation by phone call is also acceptable, but the fact that the patient survived must be recorded in the medical record.)

In the case of untraceable cases, the last date of confirmed survival prior to the loss of follow-up is considered to be the termination date.

**6.3.3. ctDNA Positive Period**

The date of surgery shall be the starting date, and when one or more cancer-related genes with genetic mutations can be identified in a blood test, the patient shall be considered　　 positive for ctDNA, and the date of the blood test shall be the time of the positive ctDNA test.

**7. completion of research**

The study is terminated when all observations have been completed and there are no abnormal findings requiring follow-up. The study will be terminated when the analysis and examination of the obtained results are completed. The results of this study will not affect the diagnosis or treatment plan of the patient in any way.

**8. discontinuation criteria**

The principal investigator or principal study investigator (hereafter referred to as the "principal investigator") shall not conduct research on individual subjects for any of the following reasons

If it is determined that continuation is not feasible, the research on the subject concerned will be terminated.

1. When a subject declines to participate in research or withdraws consent
2. Failure to meet selection/exclusion criteria
3. Death (record cause)
4. When subject follow-up is no longer possible and testing and observation can no longer be performed
5. If this entire study is terminated
6. When the person in charge of the research deems it appropriate to discontinue the research for other reasons

**9. anticipated benefits and disadvantages (side effects) associated with the study**

**9.1. the burdens and anticipated risks to the research subjects and how to minimize them**

In this study, blood samples will be drawn at the time of routine medical blood tests, with a maximum of 20 ml added per test. This will increase the amount of blood drawn, but is not expected to affect the symptoms or course of treatment of the study subjects. At the time of blood collection, the physical condition of the research subject should be carefully checked, and if the subject is unwell, blood collection should be stopped.

**9.2. overall evaluation based on expected benefits and burdens/risks**

In this study, genetic testing will be performed on patients' blood samples (and tissue samples if necessary), and the results obtained will be analyzed and discussed, but will not affect the patients' diagnosis or treatment plan in any way. Therefore, there is no benefit, burden, or risk to the participating research subjects.

**10. change, discontinuation, suspension, or termination of the research**

**10.1. changes in research**

Any changes or revisions to the research protocol for this study must be approved in advance by the Review Committee.

**10.2. termination of the study**

The study will be terminated at the end of the research period and upon completion of all genetic analyses. The principal investigator will report to the parties involved in this research that the research has been completed. The principal investigator will also report in writing to the head of the research institution to that effect and a summary of the results.

**10.3. discontinuation or suspension of research**

The person in charge of the research will consider whether or not to continue the implementation of the research if any of the following items apply

(i) When significant information is obtained regarding the quality and efficacy of the genetic testing system used in this study.

(ii) When it is judged to be extremely difficult to reach the planned number of cases due to the difficulty of incorporating the subjects.

(iii) When the Review Committee gives instructions to change the implementation plan, etc., and it is deemed difficult to accept such instructions.

The principal investigator will discontinue the research if a recommendation or instruction to discontinue is made by the Review Committee. When the decision to discontinue or suspend the research is made, regardless of the reason, the research subjects who participated in the research will be notified promptly, appropriate measures will be taken, and inspections, etc. will be conducted to ensure the safety of the research subjects. In addition, we will promptly report in writing to the President of the University, together with the reason for the suspension or interruption.

**11. method of obtaining consent**

The research investigator should submit the consent document approved by the review committee to the subject (or a surrogate if a surrogate is required).

(including a surrogate, the same applies hereinafter), provide sufficient explanation in writing and orally, and obtain the subject's free and voluntary consent in writing.

When information is obtained that may affect the subject's consent, or when changes are made to the protocol that may affect the subject's consent, the person in charge of the research shall promptly inform the subject, confirm in advance the subject's willingness to participate in the research, and obtain prior approval from the review committee to revise the consent document, etc. (see ). The consent document will be revised with the approval of the review committee in advance, and the subject's consent will be obtained again.

The consent document shall include the following

(1) The name of the research and the fact that permission to conduct said research has been obtained from the head of the research institution

(2) Name of research institution and name of principal investigator (including name of joint research institution and name of principal investigator)

Purpose and Significance of the Study

(iv) Research methods (including the purpose of use of samples and information obtained from research subjects) and duration

(5) Reason for selection as a research subject

(vi) Burden and anticipated risks and benefits to research subjects.

(vii) That consent to conduct or continue the research may be withdrawn at any time, even if it has been given.

(viii) That the research subjects will not be treated disadvantageously by refusing to consent to the implementation or continuation of the research or by withdrawing their consent.

(ix) Methods of disclosing information on research

(10) A statement that the research protocol and materials related to the research methods may be obtained or inspected upon request by the research subjects, etc., and the method of obtaining or inspecting such materials.

(11) Handling of personal information, etc. (including the method of anonymization, if any)

Methods of storage and disposal of ⑫Samples and information

⑬Status of conflicts of interest related to research, including sources of funding for research, conflicts of interest related to research at research institutions, and conflicts of interest related to research by individual researchers and others, including personal earnings

(4) Handling of results, etc. obtained through research

(xv) Responding to consultations, etc., from research subjects, etc., and other persons concerned

(iv) If there is any financial burden or gratuity to the research subject, etc., a statement to that effect and the details of such burden or gratuity.

(⑰If there is a possibility that the sample/information obtained from the research subject will be used for future research not specified at the time consent is obtained or will be provided to other research institutions, a statement to that effect and the details anticipated at the time consent is obtained.

**12. handling of personal information**

All parties involved in the research shall comply with applicable laws, regulations, and ordinances regarding the protection of personal information of research subjects. In addition, those involved shall make their utmost efforts to protect the personal information and privacy of the research subjects, and shall not divulge any personal information obtained in the course of conducting this research without justifiable reason. The same shall apply even after the person concerned has retired from his/her position.

After collecting information that can identify research subjects (initials, age, gender, medical record number, etc.), researchers will identify and manage research subjects by converting the information into a subject identification code list to be created at this facility. This list of subject identification codes will be used to ensure that personal information is not revealed when registering cases, preparing case reports, etc., and shipping specimens to other facilities. The research subject identification codes should be kept in a place that can be locked by the principal investigator.

In addition, when the principal investigators release information obtained in the research, sufficient care should be taken to ensure that the research subjects cannot be identified.

**13. the need for and availability of genetic counseling in the conduct of the study**

Since the genetic analysis in this study targets somatic gene abnormalities in cancer-related genes, it will be conducted in accordance with the "Ethical Guidelines for Life Science and Medical Research Involving Human Subjects"), which are joint guidelines issued by the three ministries of MEXT, MHLW, and METI. For concerns or consultation regarding the results of genetic analysis, referral will be made to the department in charge of genetic counseling as necessary. However, since the cost of genetic counseling for the results of genetic testing will be borne by the subject, sufficient explanation and consent must be obtained prior to enrollment in this study.

**14. statistical matters**

**14.1 Basis for setting the number of cases**

**Target number of patients:** 10 patients with colorectal cancer with liver metastases scheduled for curative resection at our institution

**Rationale: The** curative resection for liver metastases of colorectal cancer was performed at our facility during the 8-year period from January 2010 to December 2017.

The number of cases performed was 76, and it was determined that a total of 10 cases could be accumulated during the study period.

**14.2 Handling of Aborted, Missing, or Missing Measurements**

Properly stored as part of the critical data of the study and analyzed as needed.

**14.3 Analysis Methods**

The primary endpoint is to evaluate the interval between the time of diagnosis of recurrence and the time of positive ctDNA. This interval is defined as the difference between the time of diagnosis of recurrence and the time of positive ctDNA, starting from the date of surgery. The definitions of the time of diagnosis of recurrence and the time of ctDNA positivity are described in "6.3. Definition of Endpoints".

In this study, the same gene panel test by Sysmex's PSS method will be performed using ctDNA in plasma before and after surgery to measure cancer-related gene mutations. (1-1-2, Murotani 1-chome, Nishi-ku, Kobe, Hyogo, Japan) for the measurement, and will conclude an "Assay Service Agreement for ctDNA Analysis (for Research) by Plasma-Safe-SeqS Technology" with Sysmex Corporation (1-1-2, Murotani, Nishi-ku, Kobe, Hyogo, Japan).

The statistical analysis method will be the Kaplan-Meier method for DFS and OS of patients who underwent radical surgical treatment (R0/R1 resection); R2 resection cases will be omitted from the final analysis. We will also analyze the association of ctDNA status, genetic abnormalities, and clinicopathologic factors with DFS and OS using the Cox proportional hazards model. Furthermore, the association between ctDNA, genetic abnormalities, and clinicopathological factors will be analyzed using Fisher's exact test or χ-square test.

**15. storage and disposal of samples and information (including materials pertaining to information used in research) Methods of storage and disposal of samples and information (including materials pertaining to information used in research)**

**15.1. sample storage/control**

The Principal Investigator will store the tissue (FFPE) and blood specimens used in this study at this facility in a linkable anonymized form in accordance with the procedures established by this facility. The storage period will be 5 years after the date of the report of the completion of the study or 3 years after the date of the final publication of the results of the study, whichever is later. After the storage period, the data will be anonymized and disposed of in an appropriate manner.

**15.2. information storage/management**

The principal investigator shall keep documents related to the conduct of the research, etc. (copies of applications, notification documents from the President, copies of various applications and reports, consent documents, case report forms, lists of identification codes for research subjects, and other documents or records necessary to ensure the reliability of the data) in a lockable locker in the Office of the Chief of Gastroenterological Surgery. The retention period shall be until the later of the date on which the final publication of the results of the study is reported or three years have elapsed, whichever is later. After the storage period, paper media shall be shredded and discarded. Other media will be anonymized and disposed of in an appropriate manner.

**15.3 Secondary Use of Samples and Information**

There is a possibility that the samples and information collected for this study may be used in other research than this study or may be provided to other institutions (including overseas institutions) in the future when very important studies are needed, although this is not planned or foreseen at this time. For secondary use of such samples and information, a new research protocol will be prepared and reviewed and approved by the Research Ethics Review Committee. If possible, consent should be obtained after explaining again to the research subjects, but if such explanation is not possible, the research information should be made public (opt-out) in accordance with the applicable guidelines. In such cases, samples and information will not contain personally identifiable information.

**16. details and methods of reporting to the head of the research institute**

The principal investigator will report the following in this study in accordance with the rules of the relevant research institution.

　　　　　Progress of Research

　　　　　Deviations from the research protocol

　　　　　Changes to the research plan

　　　　　Report on completion of research

**Research Costs and Conflicts of Interest**

This research will be conducted under the "Assay Service Agreement for ctDNA Analysis (for Research) by Plasma-Safe-SeqS Technology" concluded by this institution with Sysmex Corporation (1-1-2, Murotani 1-chome, Nishi-ku, Kobe, Hyogo, Japan), under which this institution will entrust the measurement and analysis of tests to Sysmex Corporation, which will The implementation of the service will be carried out. The cost of the test will be paid by the research fund of the research grant.

In addition, the person in charge of this research shall report the necessary information to the Conflict of Interest Management Committee for its review and approval.

**18. details of financial burden or gratuities to research subjects, etc.**

There will be no financial burden or honorarium to the research subjects for participating in this study.

**19. compensation for damage to health**

Since this study does not involve any invasive procedures, no compensation for health hazards will be incurred, and no financial compensation will be prepared for the subject's health hazards. This point will be approved by the Ethics Committee of the institution where the research is conducted, and the subject's free and voluntary consent will be obtained in writing.

**20. attribution of research results and publication of research results**

The intellectual property rights resulting from the results of this research will be protected by an "Assay Service Agreement for ctDNA Analysis (Research) by Plasma-Safe-SeqS Technology" between Osaka Acute & Comprehensive Medical Center and Sysmex Corporation (1-1-2, Murotani, Nishi-ku, Kobe, Hyogo, Japan) , which will be implemented based on the agreement. The results of this study will be conducted in accordance with this agreement. The results obtained from this research will be presented at major relevant conferences and published in English in professional journals. In all cases, only statistically processed results will be made public, and no personal information of the research subjects will be disclosed.

**21. handling of results, etc. obtained in the research**

Regarding the disclosure of results obtained from this research, the subject's wishes shall be obtained in a consent form and appropriate action shall be taken. However, if there is a risk that disclosure of the results may harm the life, body, property, or other rights or interests of the subject or a third party, or may seriously impede the proper conduct of research work at the institution conducting the research, and if the subject's informed consent for non-disclosure has been obtained (2) The research subject's personal information shall not be disclosed, in whole or in part, in the following cases

On the other hand, if, during the course of the research, new information is obtained about the genome or other information that is being studied and is considered important for protecting the health of the subject or the subject's family, we will ask if we can disclose the information, even if the subject has checked the appropriate box on the consent form or if the subject does not wish to disclose the information, Even if the subject does not wish to disclose the information, if there are important results that we believe can be effectively addressed, we will inquire whether we can share the information with the subject after careful consideration.

**22. research implementation system**

This study will be conducted under the following structure

Research Assignee]

0 Akira Inoue, Gastroenterological Surgery (Principal Investigator)

　　　　Yujiro Nishizawa Gastroenterological Surgery

　　　　Yoshiyu Morimoto Gastroenterological Surgery

　　　　Yuki Ohsato Gastroenterological Surgery

　　　　Masahiro Hashimoto Gastroenterological Surgery

[Contact].

Osaka Acute & Comprehensive Medical Center

digestive surgery

Located at:〒 3-1 Bandaihigashi, Sumiyoshi-ku, Osaka, 558-8558− 56

Phone: 06-6692-1201

E-mail: inoue_akira@gh.opho.jp

[Inspection contractor].

Facility Name: Sysmex Corporation

Address: (1-1-2, Murotani, Nishi-ku, Kobe City, Hyogo Prefecture)

Roles and Responsibilities: Genetic analysis and reporting of specimens

**23. list of references and bibliography**

1. 2018 National Cancer Center, National Cancer Institute, Cancer Control and Information Center.

2. Colorectal Cancer Treatment Guidelines 2024 Edition Colorectal Cancer Study Group Kanehara Publishing.

3. Hasegawa K, Saiura A, Takayama T, Miyagawa S, Yamamoto J, Ijichi M, et al. Adjuvant Oral Uracil-Tegafur with Leucovorin for Colorectal Cancer Liver Metastases: A Randomized Controlled Trial. PLoS One. 2016;11(9):e0162400.

4. Portier G, Elias D, Bouche O, Rougier P, Bosset JF, Saric J, et al. Multicenter randomized trial of adjuvant fluorouracil and folinic acid compared with surgery alone after resection of colorectal liver metastases: the FFCD ACHBTH AURC 9002 trial. J Clin Oncol. 2006;24(31):4976-82.

5. Benešová L, Hálková T, Ptáčková R, Semyakina A, Menclová K, Pudil J, et al. Significance of postoperative follow-up of patients with metastatic colorectal cancer using circulating tumor DNA. world J Gastroenterol. 2019;25(48):6939-48.

6. Narayan RR, Goldman DA, Gonen M, Reichel J, Huberman KH, Raj S, et al. Peripheral Circulating Tumor DNA Detection Predicts Poor Outcomes After Liver Resection for Metastatic Colorectal Cancer. ann Surg Oncol. 2019;26(6):1824-32.

7. Kanemitsu Y, Shimizu Y, Mizusawa J, Inaba Y, Hamaguchi T, Shida D, et al. Hepatectomy Followed by mFOLFOX6 Versus Hepatectomy Alone for Liver-Only Metastatic Colorectal Cancer (JCOG0603): A Phase II or III Randomized Controlled Trial. J Clin Oncol. 2021;39(34):3789-99.

8. Muzny DM, Bainbridge MN, Chang K, Dinh HH, Drummond JA, Fowler G, et al. Comprehensive molecular characterization of human colon and rectal cancer. Nature. 2012;487(7407):330-7.

9. Nakamura Y, Taniguchi H, Ikeda M, Bando H, Kato K, Morizane C, et al. Clinical utility of circulating tumor DNA sequencing in advanced gastrointestinal Clinical utility of circulating tumor DNA sequencing in advanced gastrointestinal cancer: SCRUM-Japan GI-SCREEN and GOZILA studies. Nature Medicine. 2020.

10.Tie J, Wang Y, Tomasetti C, Li L, Springer S, Kinde I, et al. Circulating tumor DNA analysis detects minimal residual disease and predicts recurrence in patients with stage II colon cancer. Sci Transl Med. 2016;8(346):346ra92.

11.Nakamura Y, Taniguchi H, Ikeda M, Bando H, Kato K, Morizane C, et al. Clinical utility of circulating tumor DNA sequencing in advanced Nat Med. 2020;26(12):1859-64.

12.Tie J, Cohen JD, Wang Y, Christie M, Simons K, Lee M, et al. Circulating Tumor DNA Analyses as Markers of Recurrence Risk and Benefit of Adjuvant Therapy for Stage III Colon Cancer. jAMA Oncol. 2019;5(12):1710-7.

13.Tie J, Cohen JD, Wang Y, Li L, Christie M, Simons K, et al. Serial circulating tumour DNA analysis during multimodality treatment of locally advanced rectal cancer: a prospective biomarker study. Gut. 2019;68(4):663-71.

14. The Japanese Society of Pathology / ed. Regulations for the Handling of Histopathology Specimens for Genome Research and Clinical Use. Published March 01, 2019 .

15. Director, Drug Evaluation and Management Division, Pharmaceuticals and Consumer Health Bureau, Ministry of Health, Labour and Welfare. ICH Guidelines on Collection of Genomic Samples and Handling of Genomic Data . January 18, 2018 .
