# Supplementary material for: Clinical utility of circulating tumor DNA for early detection of recurrence after curative hepatectomy in patients with colorectal cancer with liver metastases: A prospective observational study protocol (CASSIOPEIA)
Source: PLoS One. 2025 Nov 20;20(11):e0335591. doi: 10.1371/journal.pone.0335591 (PMC12633885; doi:10.1371/journal.pone.0335591)
Supplement: S2 File — (DOCX) [file pone.0335591.s002.docx]

**根治切除可能な大腸癌肝転移に対して根治切除後に血液循環腫瘍DNAを**

**測定することが再発の早期発見に有用であるかを検討することを目的とした**

**単施設前向き観察研究**

**ClinicAl utility of circulating tumor DNA to detect early tumor recurrence**

**after Surgery in patients with radically reSectable lIver metastases from cOlorectal cancer: a single institute ProspEctive observatIonAl study**

**CASSIOPEIA**

**研　究　実　施　計　画　書**

作成日　　2024年10月15日　計画書案　第1版作成

**0．概要**

**0.1. 本研究の概要**

本研究では、根治切除可能な肝転移のみを有する大腸癌患者を対象に、肝転移巣に対する根治切除の前後の血液検体を用いて同一の遺伝子パネル検査を行い、遺伝子変異を有する癌関連遺伝子を測定することが再発の早期発見に有用であるかを検討する。さらに、この測定システムの本邦の実臨床での有用性ならびに保険償還を視野に入れた大規模臨床試験の立案意義について探索することを目的とする。

**
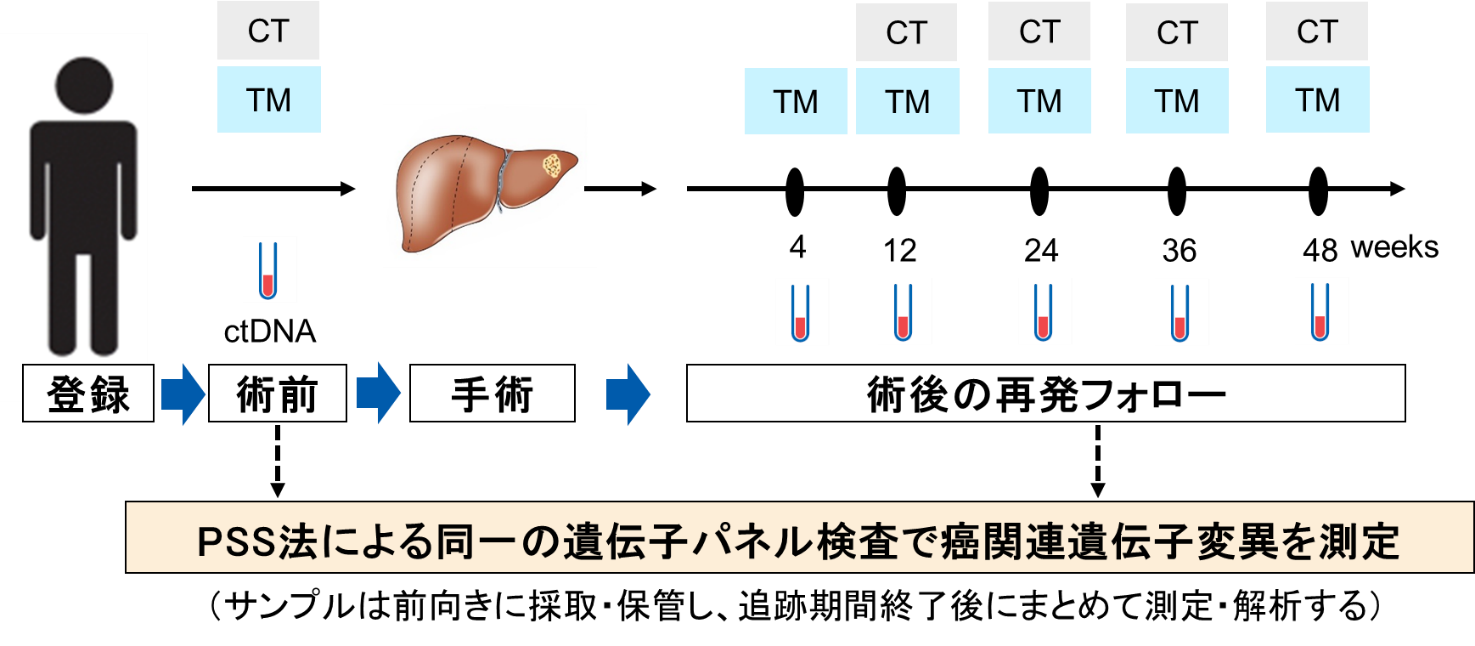
シェーマ**

**0.2. 目的**

本研究の目的は、根治切除可能な肝転移のみを有する大腸癌患者を対象に、肝転移巣に対する根治切除の術前／術後の血液検体を用いて同一の遺伝子パネル検査を行い、遺伝子変異を有する癌関連遺伝子を測定することが再発の早期発見に有用であるかを検討することである。

**0.3. 研究対象者**

**0.3.1. 選択基準**

以下の全てを満たす症例を対象とする。

①　病理組織学的に大腸癌の腺癌と診断されている。

②　原発巣の主占居部位が結腸（盲腸、結腸、直腸S状部）又は直腸と診断され、原発巣が切除されている（虫垂、肛門管癌は除く）。

③　肝転移以外の遠隔転移がなく、大腸癌肝転移に対して初回の根治切除が予定されている。

　　 （尚、全身薬物療法を行い、肝転移の根治切除が可能となった『Conversion Therapy』症例も適格とする）

④　同意取得日の年齢が20 歳以上である。

⑤　Eastern Cooperative Oncology Group（ECOG）Performance Status（PS）0 又は1である。

⑥　本研究の参加について本人より文書での同意が得られている。

**0.3.2. 除外基準**

以下のいずれかを満たす症例は除外とする。

① 肝切除の時点で活動性の重複癌が存在する。

ただし、5 年以上の無再発期間がある患者、又は局所治療により治癒したと判断される皮膚の基底細胞癌又は有棘細胞癌、表在性膀胱癌、子宮頸癌、非浸潤性乳管癌、内視鏡的治療が可能なCarcinoma in situ（上皮内癌）や粘膜内癌相当の病変、全身治療を必要としない非転移性前立腺がんを有する患者は登録可能とする。

②　妊娠中又は授乳中の女性である。

③　担当医が本研究の研究対象者として不適当と判断する。

**0.4. 評価項目（エンドポイント）**

**0.4.1. 主要評価項目（Primary endpoint）**

　　再発の診断時期と血液循環腫瘍DNA（ctDNA）の陽性時期とのインターバルを評価する。

**0.5. 目標症例数および研究期間**

**0.5.1. 目標症例数：** 当施設で根治切除を予定している肝転移のみを有する大腸癌患者10 例

**0.5.2. 登録期間：** 2024年12月1日～2025年12月1日

**0.5.3. 追跡期間：** 2024年12月1日～2026年12月1日

**0.5.4. 全研究期間：** 2024年12月1日～2027年12月1日

**0.6. 研究の方法**

**0.6.1. 研究の種類・デザイン**

本研究では前向きに症例集積してサンプルを採取・保管し、追跡期間終了後にサンプルをまとめて測定・解析する。

**0.6.2. 観察の方法**

肝転移巣に対する根治切除の術前／術後に採取した血液検体からDNAを抽出し、Plasma-Safe-Seq（PSS）法による同一の遺伝子パネル検査を行い、個々の症例における癌関連遺伝子変異を測定する。これらの測定はすべて追跡期間終了後にまとめて行う。

**0.7. 研究実施体制**

本研究は以下の体制で実施する。

【研究分担者】

〇　井上 彬　消化器外科　（研究責任者）

　　　　西沢 佑次郎　消化器外科

　　　　森本 祥悠　消化器外科

　　　　大里 祐樹　消化器外科

　　　　橋本 雅弘　消化器外科

【連絡先】

大阪急性期・総合医療センター

消化器外科

所在地：〒558-8558 大阪府大阪市住吉区万代東３丁目１−５６

電話：06-6692-1201

E-mail：inoue_akira@gh.opho.jp

**0.8. 倫理的事項**

国が定めた指針（「人を対象とする生命科学・医学系研究に関する倫理指針」）に基づき実施する。

**目　次**

0．概要 1

1．研究の背景 4

2．研究の目的 6

3．目標症例数および研究期間 6

4．研究対象者 6

5．研究の方法 6

6．評価項目（エンドポイント） 11

7．研究の終了 11

8．中止基準 11

9．研究に伴って予想される利益と不利益（副作用） 12

10．研究の変更、中止・中断、終了 12

11．同意取得方法 12

12．個人情報の取り扱い 13

13．研究実施に伴う遺伝カウンセリングの必要性と有無 13

14．統計学的事項 13

15．試料・情報（研究に用いられる情報に係る資料を含む。）の保管及び廃棄の方法 14

16．研究機関の長への報告内容及び方法 14

17．研究費用および利益相反 15

18．研究対象者等へ経済的負担又は謝礼の内容 15

19．健康被害に対する補償 15

20．研究成果の帰属と研究結果の公表 15

21．研究で得られた結果等の取扱い 15

22．研究実施体制 15

23．参考資料・文献リスト 16

**1．研究の背景**

**1.1. 結腸・直腸癌の疫学**

本邦における結腸・直腸癌の罹患率・死亡率は依然として高く、その予防や早期診断・治療法の開発は極めて重要な課題である。結腸・直腸癌の2018年の年間死亡数は、男性 27,098 人、女性 23,560 人であり、癌腫別死亡数は男性第3位、女性第1位である(1)。結腸・直腸癌の根治的切除が施行された結腸・直腸癌の5年生存率は、Stage I: 91.6%, Stage II: 84.8%, Stage IIIa: 77.7%, Stage IIIb: 60.0%, Stage IV: 18.8%である(2)。

**1.2. 結腸・直腸癌に対する標準治療**

Stage I から III の結腸・直腸癌に対しては根治的外科切除が行われる。術後の標準治療は、病理学的にリンパ節転移を有さないStage I/II では経過観察が原則であり、病理学的にリンパ節転移を有するStage III は術後補助化学療法が推奨される。遠隔転移を有するStage IV 及び再発例では、根治的外科切除が可能な場合は転移巣の外科的切除が標準治療であり、オプションとしてリスクに応じた周術期化学療法が併用される。一方で、外科的根治切除が困難な場合は全身化学療法が行われる。また、根治的外科治療後の結腸・直腸癌に対する標準的なフォローアップは、問診・診察、CT検査、腫瘍マーカー、内視鏡検査を定期的に行うことが、大腸癌治療ガイドライン（大腸癌研究会、2024年版）で推奨されている(2)。

**1.3. 肝転移を有する結腸・直腸癌に対する標準治療と臨床上の課題**

肝転移は結腸・直腸癌の遠隔転移巣として最も頻度が高い。また根治切除が可能な肝転移に対する標準治療は外科的切除である。しかし、遠隔転移巣の切除後の再発率は50～70％と高く、治療成績の向上のために術後補助化学療法の実施が議論されてきた(3, 4)。ただし、肝転移切除後に再発する患者と再発しない患者が存在し、どの患者に積極的に術後補助化学療法を行うべきかについては明らかになっていない。近年の研究結果から、再発する患者は微小残存病変（Minimal Residual Disease、以下MRD）が存在することが明らかとなり、肝転移切除後のMRDを検出し再発リスクの評価に有用であったとする研究報告(5, 6)があるものの、実臨床においては未だ確立していない。したがって、現状では術後補助化学療法により恩恵を受ける患者は一部であり、化学療法を行わなくても再発しない患者が半数近く存在する。そのような患者では副作用を伴う不必要な化学療法を受けることになり、QOL（生活の質）を大きく損なう可能性がある。以上より、個々の患者の再発リスクに応じた術後補助化学療法の実施が臨床上の課題と言える。

近年、本邦よりJCOG0603試験の結果が論文発表された(7)。この試験は大腸癌肝転移の根治切除後の患者を対象とした術後補助化学療法（mFOLFOX6療法）と手術単独を比較するランダム化試験である。その結果、主要評価項目である無病生存期間（DFS）はmFOLFOX6で有意に延長した。しかし、全生存期間（OS）では差が示されず、実臨床における解釈は未だ結論に至っていない。以上より、大腸癌治療ガイドラインおよび過去の報告では、侵襲の大きい治療である再度の肝切除を避けるという点で再発抑制自体が患者の利益となり得ることから、術後の補助化学療法を弱く推奨しているのが現状である(2, 3)。

**1.4. 大腸癌と遺伝子異常との関連**

大腸癌の発生・進展には、遺伝子異常が大きく関与していることが明らかとなっている。The Cancer Genome Atlas Network（TCGA）プロジェクト研究において、大腸癌276例の臨床サンプルを用いた全ゲノム解析が行われた(8)。その結果、大腸癌では遺伝子バリアント（変異）の多いHypermutated-typeと、多くないNonhypermutated typeに分かれることが明らかになった。前者では、ACVR2A (63%)、APC(51%)、TGFBR2(51%)、BRAF(46%)などが高頻度の遺伝子異常として検出され、後者では、APC (81%)、TP53 (60%)、KRAS (43%)、TTN (31%)が認められた。また、大腸癌の治療標的となり得るような遺伝子異常として、MYCを活性化するWNTシグナル経路やTGF-βシグナル経路に関わる遺伝子の重要性が改めて明らかとなった。さらに、IGF2, IGFR, ERBB2, ERBB3, MEK, AKT, MTORなどの遺伝子異常も認められ、これらdriver alterationを標的とした治療薬の開発が期待されている。

**1.5. 血液循環腫瘍DNAとリキッドバイオプシーについて**

近年の研究結果で、腫瘍組織から血液中に漏れ出て循環する腫瘍由来DNA（circulating tumor DNA、以下ctDNA）の存在が明らかとなった。リキッドバイオプシーとは、主に血液からctDNAを検出し、がんの診断に役立てる新しい技術であり、現在臨床開発が急速に進んでいる。リキッドバイオプシーの利点は、採血のみで行えるため、従来の腫瘍組織を用いた方法よりも簡便かつ低侵襲である。さらに、腫瘍組織の採取が困難な場合でも検査が可能なこと、繰り返し採取が可能なこと、がん関連遺伝子異常の変化が分かること、組織検査より短時間で解析できること、腫瘍の遺伝子異常の全体像が捉えられることなど、多くのメリットがある(9)。

大腸癌におけるリキッドバイオプシーの臨床応用として、①予後予測／化学療法の早期効果

判定、②分子標的薬の効果予測/獲得耐性変異の検出、③外科的治癒切除後のMRDの検出と再発リスクの評価、④がん関連遺伝子異常のプロファイリングと適切な分子標的薬の選択などがある。現在、これらを目的とした次世代シークエンサー（Next Generation Sequence、以下NGS）による遺伝子解析の研究開発が行われている。

根治切除後の大腸癌に対して、リキッドバイオプシーでctDNAを検出された症例では再発率が高いことが報告されており(10)、現在、リキッドバイオプシーで術後補助化学療法の内容を選択していく臨床試験が行われている。また、進行・再発大腸癌においてRAS遺伝子変異を有する患者は、抗EGFR抗体薬投与により利益（延命効果や腫瘍縮小）が得られない可能性が高いため、治療前にRAS野生型であることを確認しておくことが必要である。血液中のctDNAからRAS遺伝子変異の有無を評価する「OncoBEAM^TM^　RAS　CRC　キット」は、シスメックス社が開発したBEAMing法を用いた体外診断用医薬品で、本邦では2020年8月1日より保険適用となった。

　このように、リキッドバイオプシー検体に含まれるがんの遺伝子変異を、NGSなどを用いて解析することにより、がんの超早期発見や治療に用いる薬剤を、より精密かつ迅速に選択できることが期待されている(11)。本邦では、国立がん研究センター東病院を中心とした全国規模の多施設共同臨床試験の取り組み（SCRUM-Japan）が世界に先駆けて精力的に行われており、このような検査を行う医療体制が我が国でも整備されつつある。

**1.6. Plasma-Safe-Seq技術と****遺伝子パネル検査について**

Plasma-Safe-Seq（以下、PSS）技術は、医療機器メーカーであるシスメックス株式会社（兵庫県神戸市）が開発し、血漿中からctDNAを検出する新しい検査システムである。DNA分子バーコード法を用いることで、NGSの読み取りエラーを防ぐことが可能であり、NGSによる検出感度を従来の方法よりも10倍以上改善し、変異頻度（Mutant Allele Frequency、以下MAF）0.05% までの検出を可能にした。この技術を用いた臨床開発は進んでおり、230名のステージ II 大腸癌患者において根治切除後のctDNA検出の有無が再発リスクを高い精度で予測することが報告されている(10)。さらに、局所進行直腸癌やステージ III 結腸癌において根治切除後および術後補助化学療法後のctDNA検出の有無が再発リスクの評価に有用であったと報告されている(12, 13)。

PSS法による遺伝子パネル検査とは、大腸癌で変異頻度の高い14個の遺伝子に対するTarget Sequence（ターゲットシーケンス解析）を行う検査法である(10)。

本研究に先立って我々が行った検証研究では、肝転移巣の組織検体（FFPE）4例を用いてPSS法による遺伝子パネル検査を行い、ベースラインの遺伝子変異を測定したところ、遺伝子変異を有する癌関連遺伝子を4例すべてにおいて実際に同定することができた。すなわち、APC遺伝子変異は4例中3例に認めた。その他、TP53遺伝子変異を4例中2例に認め、PIK3CA遺伝子変異とNRAS遺伝子変異とSMAD4遺伝子変異をそれぞれ4例中1例ずつに認めた。

以上より、本技術を用いることで高い精度で遺伝子変異を同定できることが明らかとなり、本研究の実現可能性は極めて高いと考える。

**1.7. 本研究の意義**

本研究では、根治切除可能な肝転移のみを有する結腸・直腸癌患者を対象に、手術前後の血液検体を用いて同一の遺伝子パネル検査を行い、癌関連遺伝子変異を測定することが、肝転移に対する根治切除後の再発の早期診断に有用かを探索的に検討する。この検査システムの有用性が認められれば、今後はPMDAへの承認申請を行うための臨床試験を立案する。将来的には、個々の患者の再発リスクに応じた術後補助療法の選択的実施を評価するための臨床試験の立案も可能となる。更なる展開として、血液検体におけるがん関連遺伝子異常のプロファイリングをリアルタイムに明らかにすることで、個々の患者に適切な分子標的治療薬を選択できることが期待される。以上より、研究全体を通じて新しい診断・治療開発を行うことで、肝転移を有する結腸・直腸癌患者の利益に繋がるため、本研究の意義は極めて大きいと考える。

**2．研究の目的**

本研究の目的は、根治切除可能な肝転移のみを有する大腸癌患者を対象に、肝転移巣に対する根治切除の術前／術後の血液検体を用いて同一の遺伝子パネル検査を行い、遺伝子変異を有する癌関連遺伝子を測定することが再発の早期発見に有用であるかを検討することである。

**3．目標症例数および研究期間**

**3.1. 目標症例数：** 当施設で根治切除を予定している肝転移巣のみを有する大腸癌患者10 例

**設定根拠：** 当施設の年間症例数から算出した。

**3.2. 登録期間： 2024年12月1日～2025年12月1日**

**3.3. 追跡期間： 2024年12月1日～2026年12月1日**

**3.4. 全研究期間： 2024年12月1日～2027年12月1日**

**4．研究対象者**

**4.1. 選択基準**

以下の全てを満たす症例を対象とする。

①　病理組織学的に大腸癌の腺癌と診断されている。

②　原発巣の主占居部位が結腸（盲腸、結腸、直腸S状部）又は直腸と診断され、原発巣が切除されている（虫垂、肛門管癌は除く）。

③　肝転移以外の遠隔転移がなく、大腸癌肝転移に対して初回の根治切除が予定されている。

　　 （尚、全身薬物療法を行い、肝転移の根治切除が可能となった『Conversion Therapy』症例も適格とする）

④　同意取得日の年齢が20 歳以上である。

⑤　Eastern Cooperative Oncology Group（ECOG）Performance Status（PS）0 又は1である。

⑥　本研究の参加について本人より文書での同意が得られている。

**4.2. 除外基準**

以下のいずれかを満たす症例は除外とする。

① 肝切除の時点で活動性の重複癌が存在する。

ただし、5 年以上の無再発期間がある患者、又は局所治療により治癒したと判断される皮膚の基底細胞癌又は有棘細胞癌、表在性膀胱癌、子宮頸癌、非浸潤性乳管癌、内視鏡的治療が可能なCarcinoma in situ（上皮内癌）や粘膜内癌相当の病変、全身治療を必要としない非転移性前立腺がんを有する患者は登録可能とする。

②　妊娠中又は授乳中の女性である。

③　担当医が本研究の研究対象者として不適当と判断する。

**5．研究の方法**

**5.1. 本研究の概要**

本研究の概要のシェーマを下記の図に示す。本研究の対象は、根治切除可能な肝転移のみを有する大腸癌患者である。肝転移巣に対する根治切除術の術前／術後の血液検体を採取し、PSS法による同一の遺伝子パネル検査を行い、癌関連遺伝子変異を測定する。サンプルは前向きに採取・保管し、追跡期間終了後に後ろ向きにサンプルをまとめて測定・解析する。本研究の目的は、肝転移巣に対する根治切除の術前／術後の血液検体を用いて同一の遺伝子パネル検査を行い、癌関連遺伝子変異を測定することが再発の早期発見に有用であるかを検討することである。

**シェーマ
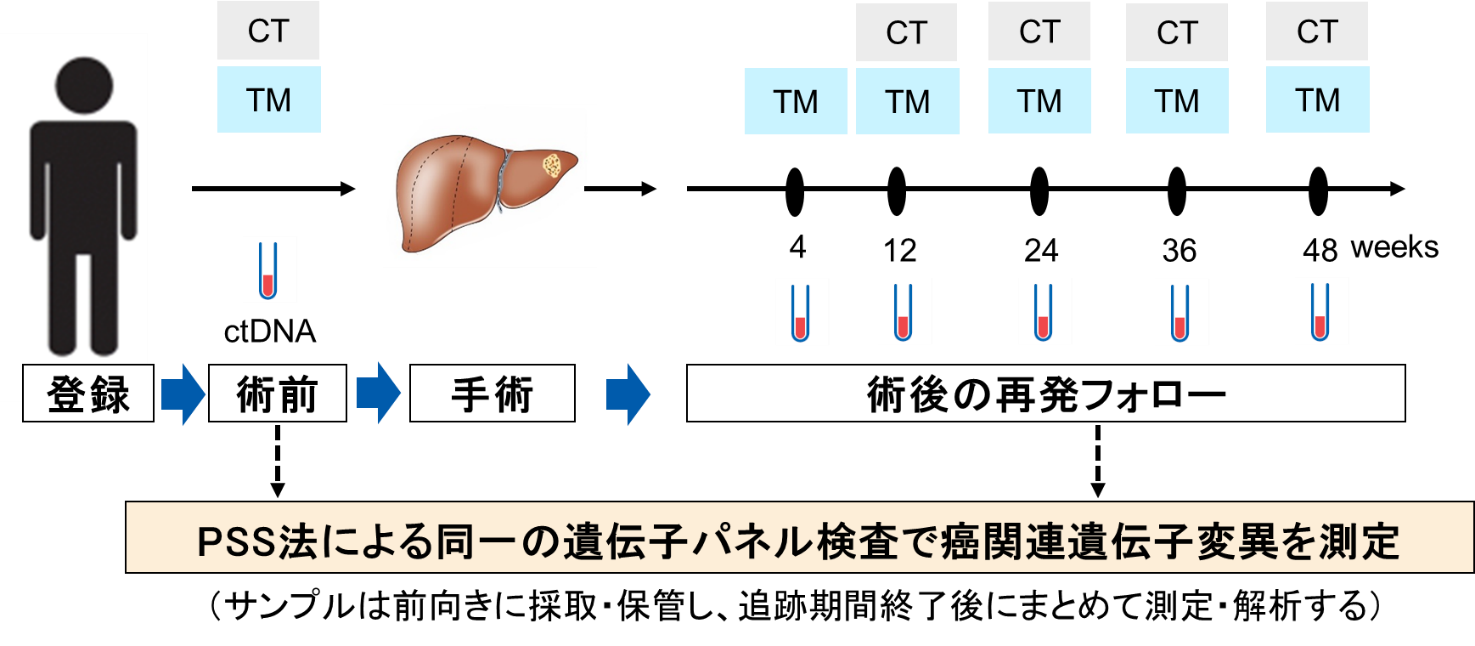
**

**5.2. 研究の種類・デザイン**

本研究では前向きに症例集積してサンプルを採取・保管し、追跡期間終了後にサンプルをまとめて測定・解析する。

**5.3. 観察の方法**

肝転移巣に対する根治切除の術前／術後に採取した血液検体からDNAを抽出し、Plasma-Safe-Seq（PSS）法による同一の遺伝子パネル検査を行い、個々の症例における癌関連遺伝子変異を測定する。これらの測定はすべて追跡期間終了後にまとめて行う。

**5.4. 研究対象者の登録方法**

　　　研究責任者及び研究分担者は、文書にて同意を取得した研究対象者に対して適格性の確認を行い、適格とされた方を研究対象者として本研究への登録を行う。

**5.5. 研究の対象とする医薬品・医療機器、治療法等の情報や使用方法など**


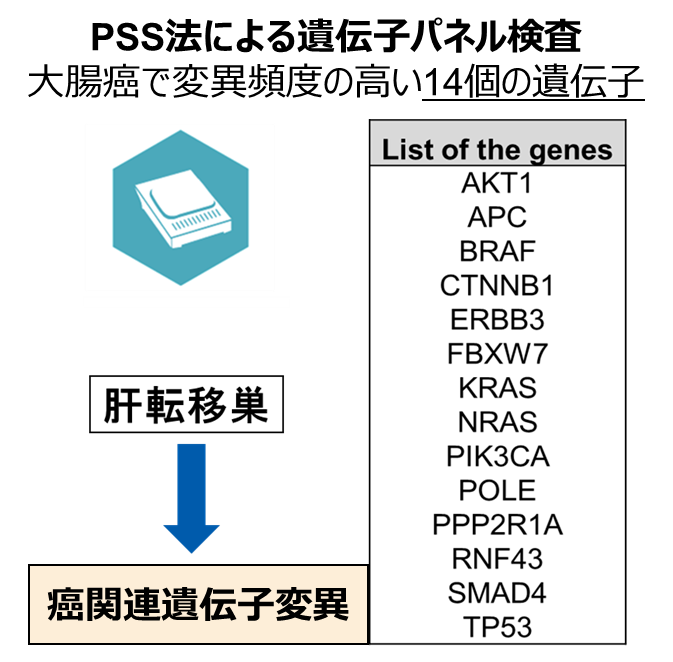
本研究に先立って我々が行った検証研究では、肝転移巣の組織検体（FFPE）を用いてシスメックス社のPSS法による遺伝子パネル検査で遺伝子解析を行った。PSS法による遺伝子パネル検査とは、大腸癌で変異頻度の高い14個の遺伝子に対するTarget Sequence（ターゲットシーケンス解析）を行う検査法である(10)。本研究に先立って我々が行った検証研究では、個々の患者の腫瘍組織（肝転移巣）および血液検体を用いて、同一の遺伝子パネル検査を行い、癌関連遺伝子変異を測定した。

本研究に先立って我々が行った検証研究では、肝転移巣の組織検体（FFPE）4例を用いてPSS法による遺伝子パネル検査を行い、ベースラインの遺伝子変異を測定したところ、遺伝子変異を有する癌関連遺伝子変異を4例すべてにおいて実際に同定することができた（表1）。すなわち、APC遺伝子変異は4例中3例に認めた。その他、TP53遺伝子変異を4例中2例に認め、PIK3CA遺伝子変異とNRAS遺伝子変異とSMAD4遺伝子変異をそれぞれ4例中1例ずつに認めた。

(表1)

| **患者情報** | **遺伝子名 \| 塩基配列変異 \| 変異頻度** |
| --- | --- |
| 67歳　男性  横行結腸癌　異時性肝転移 | APC \| c.4037C>G \| 13.699% APC \| c.847C>T \| 23.37%  TP53 \| c.742C>T \| 30.832% |
| 70歳　男性  直腸癌　同時性肝転移 | APC \| c.4057G>T \| 5.515% APC \| c.637C>T \| 5.283% |
| 78歳　男性  直腸癌　同時性肝転移 | APC \| c.4135G>T \| 16.292% APC \| c.904C>T \| 37.215% PIK3CA \| c.1633G>A \| 17.922% |
| 45歳　女性  S状結腸癌　異時性肝転移 | NRAS \| c.182A>T \| 42.024% SMAD4 \| c.1082G>A \| 68.634% TP53 \| c.422G>A \| 70.527% |

**5.6. 調査項目及び臨床検査**

**5.6.1. 研究対象者背景**

研究対象者のイニシャル、施設症例番号、性別、生年月又は年齢（登録時）を収集する。

また、原疾患については以下の項目等を調査する。

1) 全身状態

・ Performance status（ECOG）

・ 合併症、既往歴、内服歴、喫煙歴

2) 原発臓器に関する情報

・ 原発巣主占居部位

・ 初回診断日

・ 病理学的組織型

・ 原発巣切除後のUICC 第8 版における臨床病期分類

3) 日常診療で撮像された画像診断

・ CT 検査

・ MRI 検査

・ PET-CT 検査

・ 消化管内視鏡検査

4) 日常診療で検査された腫瘍マーカー、遺伝子変異の情報

・ 腫瘍マーカー（CEA, CA19-9, p53）

・ 遺伝子変異（RAS, BRAF, MSI, MMR, HER2, TMB, NTRK, RET, その他、遺伝子パネル検査で得られた遺伝子変異の情報）

5) 前治療歴

・ 治癒切除前の化学放射線療法又は化学療法歴

・ 初回手術（原発巣切除術）の情報

**5.6.2. 登録後臨床経過**

本研究の登録後に以下の項目等を調査する。

1) 肝転移巣に対する切除後の病理診断

・ 手術時のUICC 第8 版及び大腸癌取り扱い規約第9 版における病理学的分類

・ 病理組織所見（病理学的組織型、壁深達度、浸潤増殖様式、リンパ管侵襲、静脈侵襲、簇出、近位及び遠位切離断面、深部切離断面における癌浸潤露出の有無、多発癌、リンパ節転移、遠隔臓器転移、癌遺残度、根治度等）

2) 日常診療で撮像された画像診断

・ CT 検査

1. 手術日を起算日として、48週までは12週毎（±2週を許容、同一曜日可）に行う。
2. 本観察研究に関連した試験に登録された場合、それぞれの試験に規定された間隔で画像評価を実施する。

・ MRI 検査

・ PET-CT 検査

・ 消化管内視鏡検査

3) 日常診療で検査された腫瘍マーカー

・ CEA, CA19-9

1. 手術日を起算日として、術後4週及び48週までは12週毎（±2週を許容、同一曜日可）に行う。
2. 本観察研究に関連した試験に登録された場合、それぞれの試験に規定された間隔で血液検査を実施する

4) 臨床経過

・ 術式

・ 周術期合併症（Clavien Dindo Grade2 以上）

・ 周術期治療を受けた場合はレジメン、治療開始日、最終投与日

・ 再発日

・ 最終生存確認日

・ 再発後の治療内容

**5.6.3. 組織試料**

本研究では、必要に応じて組織試料を解析することも想定している。当施設において大腸癌の肝転移に対して外科的切除された腫瘍組織（肝転移巣）から作成されたFFPE（ホルマリン固定パラフィン包埋）検体を用いる。解析に必要な検体量（25 mm^2^、10μm厚、スライド8枚、腫瘍含有量が20％以上）を検査施設に送付する。必要に応じて、この腫瘍組織の検体を用いて遺伝子検査を行う。さらに、アウトカムや他の臨床病理学的・分子生物学的特徴との関連を評価する。これらの測定・解析はすべて追跡期間終了後にまとめて行う。

試料の取扱いは、ゲノム研究用・診療用病理組織検体取扱い規程に準ずる(14)。

**5.6.4. 血液検体**

本研究では、研究用に血液を手術前（術前4週間以内、同一曜日可）、術後4週（±1週を許容、同一曜日可）及び術後12週毎（±2週を許容、同一曜日可）に術後48週目まで、それぞれ1回あたり20mlを採取する。当施設においてプロトコールに従い遠心分離を行い血漿を採取し、超低温フリーザー（－70℃以下）で保管する。（詳細はCASSIOPEIA検体採取マニュアルを参照）。再発と診断された時点で血液検体を採取し、それ以降の研究用の血液検体の採取は終了とする。一方、術後48週を超えた時期に再発した場合は、その時点で血液検体を採取する。規定の採血時期に採取できなかった症例等については、責任医師の判断にて規定の採血時期以外でも研究用血液検体採取を行うことがある。その場合でも日常診療で施行される検査の際に必要量を上乗せして採取する。CEA、CA19-9の採血や画像検査等の検査は、本研究で規定する観察時期に実施する。上記また、R2切除となった症例は最終解析から省くため、術後4週以降の研究用採血は行わない。さらに、研究用採血の2週間以内に輸血歴がある場合には検査結果に影響を及ぼすため、検査日を変更するか、許容日を外れる場合には、該当時の採血は行わないこととする。

本研究に登録されたのち、本研究に関連した試験に登録された場合は、それぞれの試験で規定される日程でも検査を行う。採取した血液は当施設からシスメックス株式会社（神戸市）に送付する。血漿については、PSS法による遺伝子パネル検査を用いてctDNA 解析が行われる。これらの測定・解析はすべて追跡期間終了後にまとめて行う。

試料の取扱いは、ゲノム試料の収集及びゲノムデータの取扱いに関するガイドラインに準ずる(15)。

**5.7. 本研究の検査項目及び検査スケジュール**

|  | 登録前 | 手術日 | 術後48週まで | | | | |
| --- | --- | --- | --- | --- | --- | --- | --- |
|  |  |  | （～1年） | | | | |
| 術後経過年月 |  |  | 4 週 | 12週 | 24週 | 36週 | 48週 |
| 許容範囲 |  |  | ±1 週 | ±2 週 | ±2 週 | ±2 週 | ±2 週 |
| 同意取得 | ○ |  |  |  |  |  |  |
| 選択基準/除外基準 | ○ |  |  |  |  |  |  |
| ECOG PS | ○ |  |  |  |  |  |  |
| 病変評価 | ○ |  |  |  |  |  |  |
| 手術・病理所見 |  |  | ○++ |  |  |  |  |
| 前治療歴 | ○ |  |  |  |  |  |  |
| CEA、CA19-9 | ○ |  | ● | ●+ | ●+ | ●+ | ●+ |
| 転帰 |  |  | ▲ | ▲ | ▲ | ▲ | ▲ |
| 胸腹骨盤部CT | ○ |  |  | ●+ | ●+ | ●+ | ●+ |
| 全大腸内視鏡検査^1)^ | ○ |  |  |  |  |  | ＊ |
| 腫瘍組織 |  | ○+ |  |  |  |  |  |
| ctDNA用採血 |  | ◎ | ● | ●+ | ●+ | ●+ | ●+ |

CEA、CA19-9 などの血液検査、胸腹骨盤部CT 検査、全大腸内視鏡検査は日常診療で施行された結果を収集する。

1) *：術前又は術後に全大腸の観察ができなかった場合には登録後1 年以内に施行する。その後は腫瘍性病変を認める場合は1年毎、認めない場合は3-5年毎に検査する。

〇：登録前4週以内（同一曜日含む）

〇+：腫瘍組織はFFPEの薄切スライドを当施設より検査施設へ送付する。

〇++：手術・病理結果は結果が分かり次第、当施設で保管する。

◎：手術前4週以内（同一曜日含む）、但し術前治療が施行されている場合は術前治療終了後から手術前までとする。

●：規定日±1週（同一曜日含む）を許容する

●+：規定日±2週（同一曜日含む）を許容する（6.3.1.で定義した「再発」と診断された時点で、それ以降の ctDNA用採血は終了とする。）

▲：この期間に収集する

**5.8. 研究終了後の対応**

本研究の終了後は、得られた結果を解析・検討した上で、速やかに次の研究へ向けた準備を行う。尚、本研究の結果は患者の診断・治療方針には一切影響しない。

**5.9. 他の機関等の試料・情報の利用**

　　 なし

**6．評価項目（エンドポイント）**

**6.1. 主要評価項目（Primary endpoint）**

再発の診断時期と血液循環腫瘍DNA（ctDNA）の陽性時期とのインターバルを評価する。ctDNAの陽性とは血液検査で遺伝子変異を有する癌関連遺伝子を1つ以上同定できた場合と定義する。

**6.2. 副次的評価項目（Secondary endpoint）**

手術前後の血液検体における癌関連遺伝子変異の一致率および、その遺伝子プロファイリングを評価する。また、肝転移巣の組織検体における病理組織学的な評価を行い、必要に応じて肝転移巣の組織検体における癌関連遺伝子についても解析を行う。また、臨床アウトカムとして、無病生存期間（Disease-free Survival：DFS）、全生存期間（Overall survival: OS）、再発時のctDNA陽性の有無などを設定し、これらのアウトカム間での相関や他の臨床病理学的・分子生物学的特徴との関連について探索的に解析を行う。

**6.3. エンドポイントの定義**

**6.3.1. 無病生存期間（Disease free survival: DFS）**

・ 手術日を起算日とし、再発と診断された日、又はあらゆる原因による死亡日のうち早い方までの期間。「再発」は、画像診断に基づいて判断されるものとし、その画像検査を行った検査日を再発時期とする。画像診断によらない病状の増悪や腫瘍マーカーの上昇のみの期間は再発とせず、画像診断で再発を確認した検査日をもって再発の診断時期とする。

・ 再発の画像診断については「画像上疑い」の検査日ではなく、「確診」が得られた画像検査の「検査日」をもってイベントとする。

・ 再発と判断されていない生存例では、最終生存確認日をもって打ち切りとする（電話連絡による生存確認も可。ただし生存確認を行ったことを診療録に記録すること）。

・ 再発の確定診断が生検病理診断による場合、画像上再発と診断し得ず生検病理診断によって再発と診断した場合は生検日をもってイベントとする。

・ 二次癌（異時性重複癌、異時性多発癌を含む）の発生はイベントとも打ち切りともせず、他のイベントが観察されるまでDFS とする。

・ 病理所見で癌遺残度がR2 となった場合は、主解析の対象外とする（「14.3. 解析方法」参照）。

**6.3.2. 全生存期間（Overall survival: OS）**

・ 手術日を起算日とし、あらゆる原因による死亡日までの期間。

・ 生存例では最終生存確認日をもって打ち切りとする（電話連絡による生存確認も可。ただし生存確認を行ったことを診療録に記録すること）。

・ 追跡不能例では追跡不能となる以前で生存が確認されていた最終日をもって打ち切りとする。

**6.3.3. ctDNAの陽性時期**

・　手術日を起算日とし、血液検査で遺伝子変異を有する癌関連遺伝子を1つ以上同定できた場合を　　ctDNAの陽性と判断し、その血液検査の検査日をもってctDNAの陽性時期とする。

**7．研究の終了**

すべての観察が完了し、追跡の必要な異常所見がみられない事を確認した時点を、その被験者における研究の終了とする。また、得られた結果に対する解析・検討が完了した時点を研究の終了とする。尚、本研究の結果は患者の診断・治療方針には一切影響を及ぼさない。

**8．中止基準**

研究責任者または研究分担者（以下、研究担当者）は、次に挙げる理由で個々の被験者について研究

継続が不可能と判断した場合には、当該被験者についての研究を中止する。

1. 被験者から研究参加の辞退の申し出や同意の撤回があった場合
2. 選択/除外基準への不適合
3. 死亡（原因を記録する）
4. 被験者フォローアップが不可能となり、検査および観察が出来なくなった場合
5. 本研究全体が中止された場合
6. その他の理由により、研究担当者が研究の中止が適当と判断した場合

**9．研究に伴って予想される利益と不利益（副作用）**

**9.1. 研究対象者に生じる負担と予想されるリスク、それらを最小化する方法**

本研究は、日常診療の血液検査時に1回につき最大20mlを上乗せして採血を行う。そのため、採血量が増加するが、研究対象者の症状や治療経過に影響を与えないものと考えられる。採血時には、研究対象者の体調をよく確認し、不調であれば採血を中止する。

**9.2. 予想される利益と負担・リスクを踏まえた総合評価**

本研究では、患者の血液検体（必要に応じて組織検体）を用いて遺伝子検査を行い、得られた結果の解析・検討を行うが、患者の診断・治療方針には一切影響を及ぼさない。したがって、参加した研究対象者に利益や負担・リスクはない。

**10．研究の変更、中止・中断、終了**

**10.1. 研究の変更**

本研究の研究実施計画書の変更または改訂を行う場合は、あらかじめ審査委員会の承認を必要とする。

**10.2. 研究の終了**

研究期間の終了及びすべての遺伝子解析の完了をもって、研究を終了する。研究責任者は、研究が終了したことを本研究の関係者に報告する。また、研究責任者は研究機関の長に対して、その旨及び結果の概要を文書で報告する。

**10.3. 研究の中止、中断**

研究担当者は、以下の事項に該当する場合は、研究実施継続の可否を検討する。

①　本研究で用いる遺伝子検査システムの品質、有効性に関する重大な情報が得られたとき。

②　被験者の組み入れが困難で、予定症例数に達することが極めて困難であると判断されたとき。

③　審査委員会により実施計画等の変更の指示があり、これを受入れることが困難と判断されたとき。

研究責任者は、審査委員会により中止の勧告あるいは指示があった場合は、研究を中止する。また、研究の中止または中断を決定した時は、理由の如何を問わず、本研究に参加した研究対象者に速やかにその旨を通知し、適切な措置を講じるとともに、研究対象者の安全を確保するための検査等を実施する。また、速やかに総長にその理由とともに文書で報告する。

**11．同意取得方法**

研究担当者は、審査委員会で承認の得られた同意説明文書を被験者（代諾者が必要な場合は

代諾者を含む、以下同じ）に渡し、文書および口頭による十分な説明を行い、被験者の自由意思による同意を文書で取得する。

研究担当者は、被験者の同意に影響を及ぼす情報が得られたときや、被験者の同意に影響を及ぼすような実施計画等の変更が行われるときは、速やかに被験者に情報提供し、研究に参加するか否かについて被験者の意思を予め確認するとともに、事前に審査委員会の承認を得て同意説明文書等の改訂を行い、被験者の再同意を得ることとする。

同意説明文書には、以下の内容を含むものとする。

①研究の名称及び当該研究の実施について研究機関の長の許可を受けている旨

②研究機関の名称及び研究責任者の氏名（共同研究機関の名称及び研究責任者の氏名を含む）

③研究の目的及び意義

④研究の方法（研究対象者から取得された試料・情報の利用目的を含む）及び期間

⑤研究対象者として選定された理由

⑥研究対象者に生じる負担並びに予測されるリスク及び利益

⑦研究が実施又は継続されることに同意した場合であっても随時これを撤回できる旨

⑧研究が実施又は継続されることに同意しないこと又は同意を撤回することによって研究対象者等が不利益な取扱いを受けない旨

⑨研究に関する情報公開の方法

⑩研究対象者等の求めに応じて、研究計画書及び研究の方法に関する資料を入手又は閲覧できる旨並びにその入手又は閲覧の方法

⑪個人情報等の取扱い（匿名化する場合にはその方法を含む）

⑫試料・情報の保管及び廃棄の方法

⑬研究の資金源等、研究機関の研究に係る利益相反及び個人の収益等、研究者等の研究に係る利益相反に関する状況

⑭研究により得られた結果等の取扱い

⑮研究対象者等及びその関係者からの相談等への対応

⑯研究対象者等に経済的負担又は謝礼がある場合には、その旨及びその内容

⑰研究対象者から取得された試料・情報について、同意を受ける時点では特定されない将来の研究のために用いられる可能性又は他の研究機関に提供する可能性がある場合には、その旨と同意を受ける時点において想定される内容

**12．個人情報の取り扱い**

研究に関わる関係者は、研究対象者の個人情報保護について、適応される法令、条例を遵守する。また、関係者は研究対象者の個人情報及びプライバシー保護に最大限の努力を払い、本研究を行う上で知り得た個人情報を正当な理由なく漏らしてはならない。関係者がその職を退いた後も同様とする。

研究者等は、研究対象者を特定できる情報（イニシャル、年齢、性別、カルテ番号等）を収集した上で、当施設にて作成する被験者識別コードリストに変換して研究対象者を識別・管理する。症例登録や症例報告書等の作成、また他施設への検体の発送等の際には、この被験者識別コードリストを用いて個人情報が分からないように行う。なお、研究対象者識別コードは研究責任者が施錠できる場所に保管する。

　　また、研究責任者等が研究で得られた情報を公表する際には、研究対象者が特定できないよう十分に配慮する。

**13．研究実施に伴う遺伝カウンセリングの必要性と有無**

本研究における遺伝子解析は、がん関連遺伝子の体細胞遺伝子異常を対象とするため、文部科学省、厚生労働省、経済産業省の三省合同指針である「人を対象とする生命科学・医学系研究に関する倫理指針」）に基づき実施する。遺伝子解析結果に関しての不安や相談に関しては、必要に応じて遺伝カウンセリング担当部署へ紹介する。ただし、遺伝子検査の結果に対する遺伝カウンセリングに掛かる費用は被験者様の自己負担となるため、本研究への登録前に十分な説明と同意を得る必要がある。

**14．統計学的事項**

**14.1. 症例数の設定根拠**

**目標症例数：** 当施設で根治切除を予定している肝転移巣のみを有する大腸癌患者10 例

**設定根拠：** 2010年1月～2017年12月までの8年間で、当施設で大腸癌の肝転移に対して治癒切除を施

行した症例は76例であり、研究期間内で計10例の症例が集積可能と判断した。

**14.2. 中止・脱落・欠測値の取り扱い**

研究の重要なデータの一部として適切に保管し、必要に応じて解析を行う。

**14.3. 解析方法**

主要評価項目（Primary endpoint）は、再発の診断時期とctDNAの陽性時期とのインターバルを評価することである。このインターバルの定義は、手術日を起算日とした再発の診断時期とctDNAの陽性時期との期間の差である。再発の診断時期とctDNAの陽性時期の定義は、「6.3. エンドポイントの定義」に記載した。

本研究では、手術前後の血漿中のctDNAを用いてシスメックス社のPSS法による同一の遺伝子パネル検査を行い、癌関連遺伝子変異を測定する。測定については、シスメックス株式会社（兵庫県神戸市西区室谷1丁目1番地の2）と「Plasma-Safe-SeqS技術によるctDNA解析（研究用）アッセイサービス契約書」を締結し、測定を委託する。

また、統計解析方法としては、カプランマイヤー法を用いて根治的外科治療（R0/R1切除）が行われた症例のDFS、及びOSの解析を行う。R2切除症例については最終解析から省く予定である。また、ctDNA statusや遺伝子異常、臨床病理学的因子とDFS及びOSとの関連について、コックス比例ハザードモデルを用いて解析を行う。さらにctDNAや遺伝子異常、臨床病理学的因子の関連について、Fisherの正確検定又はχ二乗検定等で解析を行う。

**15．試料・情報（研究に用いられる情報に係る資料を含む。）の保管及び廃棄の方法**

**15.1. 試料の保管/管理**

研究責任者は、本研究で用いる組織検体（FFPE）および血液検体を当施設の定める手順書に従って連結可能匿名化して、当施設で保管する。保管期間は、当該研究の終了について報告された日から5年を経過した日又は当該研究の結果の最終の公表について報告された日から3年を経過した日のいずれか遅い日までの期間とする。保管期間終了後は、匿名化のうえ適切な方法で廃棄する。

**15.2. 情報の保管/管理**

研究責任者は、研究等の実施に係わる文書（申請書類の控え、総長からの通知文書、各種申請書・報告書の控、同意文書、症例報告書、研究対象者識別コードリストその他データの信頼性を保証するのに必要な書類又は記録等）を消化器外科医局の鍵のかかるロッカーに保管する。保管期間は、当該研究の結果の最終の公表について報告された日から3年を経過した日のいずれか遅い日までの期間とする。保管期間終了後は、紙媒体に関してはシュレッダーで裁断し廃棄する。その他媒体に関しては、匿名化のうえ適切な方法で廃棄する。

**15.3. 試料及び情報の二次利用**

本研究のために集められた試料及び情報を、現時点では、計画・予測されていないものの、将来非常に重要な検討が必要となる場合に本研究とは別の研究で利用する可能性、又は他機関（海外を含む）に提供される可能性がある。こうした試料及び情報の二次利用に関しては、改めて研究計画書を作成し、研究倫理審査委員会で審査・承認を受ける。また、可能な限り研究対象者に改めて説明の上、同意を取得するが、説明できない場合には、該当する指針に基づき研究の情報の公開等（オプトアウト）を行う。この際も、試料及び情報に個人を特定できる情報を含むことはない。

**16．研究機関の長への報告内容及び方法**

研究責任者は当該研究機関のルールに則り、本研究における以下の報告を行う。

　　　　　・研究の進捗状況

　　　　　・研究計画書からの逸脱

　　　　　・研究計画書の変更

　　　　　・研究終了の報告

**17．研究費用および利益相反**

本研究は、当施設がシスメックス株式会社（兵庫県神戸市西区室谷1丁目1番地の2）と「Plasma-Safe-SeqS技術によるctDNA解析（研究用）アッセイサービス契約書」を締結し、当施設がシスメックス株式会社に検査の測定・解析を委託し、シスメックス株式会社が実施する。検査費用は、研究助成金の研究費により支払われる。

また、本研究の研究担当者は、利益相反マネジメント委員会に必要事項を申告し、その審査と承認を得るものとする。

**18．研究対象者等へ経済的負担又は謝礼の内容**

本研究に参加することによる研究対象者への経済的負担や謝礼は一切発生しない。

**19．健康被害に対する補償**

本研究は侵襲を伴わないため、健康被害に対する補償が生じることはなく、被験者の健康被害に対する金銭的な補償は準備しない。この点を研究実施施設の倫理委員会の承認を得るとともに、被験者の自由意思による同意を文書で取得する。

**20．研究成果の帰属と研究結果の公表**

本研究で得られた成果による知的財産権については、大阪急性期・総合医療センターとシスメックス株式会社（兵庫県神戸市西区室谷1丁目1番地の2）との間に「Plasma-Safe-SeqS技術によるctDNA解析（研究用）アッセイサービス契約書」を締結し、それに基づいて実施する。また、本研究で得られた結果は、主要な関連学会で発表し、専門学術誌に英文論文として公表する予定である。いずれの場合においても公表する結果は統計的な処理を行ったものだけとし、研究対象者の個人情報は一切公表しない。

**21．研究で得られた結果等の取扱い**

　本研究により得られた結果の開示については、被験者の希望を同意書で取得し、適切に行うこととする。ただし、結果を開示することにより被験者若しくは第三者の生命、身体、財産その他の権利利益を害するおそれ又は当該研究を行う機関の研究業務の適正な実施に著しい支障を及ぼすおそれがあり、かつ、開示しないことについて被験者のインフォームド・コンセントを受けている場合には、全部又は一部を開示しないこととする。

一方、研究の途中で、今回調べているゲノムなどについて新たな情報が得られ、被験者や被験者の家族の健康を守る上で重要と考えられることが判明した場合、その情報について開示を希望されていて同意書にある該当部分にチェックされている場合および、開示を希望されない場合でも、重要な結果が判明し、かつそれに有効な対処法があると考えられる場合には、慎重に検討した上で被験者にその内容を伝えて良いか問い合わせすることとする。

**22．研究実施体制**

本研究は以下の体制で実施する。

【研究分担者】

〇　井上 彬　消化器外科　（研究責任者）

　　　　西沢 佑次郎　消化器外科

　　　　森本 祥悠　消化器外科

　　　　大里 祐樹　消化器外科

　　　　橋本 雅弘　消化器外科

【連絡先】

大阪急性期・総合医療センター

消化器外科

所在地：〒558-8558 大阪府大阪市住吉区万代東３丁目１−５６

電話：06-6692-1201

E-mail：inoue_akira@gh.opho.jp

【検査の委託先】

施設名：シスメックス株式会社

住所：（兵庫県神戸市西区室谷1丁目1番地の2）

役割及び責任：検体の遺伝子解析および報告

**23．参考資料・文献リスト**

1. 2018 国立研究開発法人国立がん研究センターがん対策情報センター.

2. 大腸癌治療ガイドライン 2024 年版 大腸癌研究会 金原出版.

3. Hasegawa K, Saiura A, Takayama T, Miyagawa S, Yamamoto J, Ijichi M, et al. Adjuvant Oral Uracil-Tegafur with Leucovorin for Colorectal Cancer Liver Metastases: A Randomized Controlled Trial. PLoS One. 2016;11(9):e0162400.

4. Portier G, Elias D, Bouche O, Rougier P, Bosset JF, Saric J, et al. Multicenter randomized trial of adjuvant fluorouracil and folinic acid compared with surgery alone after resection of colorectal liver metastases: FFCD ACHBTH AURC 9002 trial. J Clin Oncol. 2006;24(31):4976-82.

5. Benešová L, Hálková T, Ptáčková R, Semyakina A, Menclová K, Pudil J, et al. Significance of postoperative follow-up of patients with metastatic colorectal cancer using circulating tumor DNA. World J Gastroenterol. 2019;25(48):6939-48.

6. Narayan RR, Goldman DA, Gonen M, Reichel J, Huberman KH, Raj S, et al. Peripheral Circulating Tumor DNA Detection Predicts Poor Outcomes After Liver Resection for Metastatic Colorectal Cancer. Ann Surg Oncol. 2019;26(6):1824-32.

7. Kanemitsu Y, Shimizu Y, Mizusawa J, Inaba Y, Hamaguchi T, Shida D, et al. Hepatectomy Followed by mFOLFOX6 Versus Hepatectomy Alone for Liver-Only Metastatic Colorectal Cancer (JCOG0603): A Phase II or III Randomized Controlled Trial. J Clin Oncol. 2021;39(34):3789-99.

8. Muzny DM, Bainbridge MN, Chang K, Dinh HH, Drummond JA, Fowler G, et al. Comprehensive molecular characterization of human colon and rectal cancer. Nature. 2012;487(7407):330-7.

9. Nakamura Y, Taniguchi H, Ikeda M, Bando H, Kato K, Morizane C, et al. Clinical utility of circulating tumor DNA sequencing in advanced gastrointestinal cancer: SCRUM-Japan GI-SCREEN and GOZILA studies. Nature Medicine. 2020.

10.Tie J, Wang Y, Tomasetti C, Li L, Springer S, Kinde I, et al. Circulating tumor DNA analysis detects minimal residual disease and predicts recurrence in patients with stage II colon cancer. Sci Transl Med. 2016;8(346):346ra92.

11.Nakamura Y, Taniguchi H, Ikeda M, Bando H, Kato K, Morizane C, et al. Clinical utility of circulating tumor DNA sequencing in advanced gastrointestinal cancer: SCRUM-Japan GI-SCREEN and GOZILA studies. Nat Med. 2020;26(12):1859-64.

12.Tie J, Cohen JD, Wang Y, Christie M, Simons K, Lee M, et al. Circulating Tumor DNA Analyses as Markers of Recurrence Risk and Benefit of Adjuvant Therapy for Stage III Colon Cancer. JAMA Oncol. 2019;5(12):1710-7.

13.Tie J, Cohen JD, Wang Y, Li L, Christie M, Simons K, et al. Serial circulating tumour DNA analysis during multimodality treatment of locally advanced rectal cancer: a prospective biomarker study. Gut. 2019;68(4):663-71.

14.一般社団法人日本病理学会／編. ゲノム研究用・診療用病理組織検体取扱い規程. 2019年03月01日発行.

15.厚生労働省医薬・生活衛生局医薬品審査管理課長. ICHガイドライン　ゲノム試料の収集及びゲノムデータの取扱いに関するガイドライン. 2018年1月18日.
