# Supplementary material for: Clinical utility of circulating tumor DNA for early detection of recurrence after curative hepatectomy in patients with colorectal cancer with liver metastases: A prospective observational study protocol (CASSIOPEIA)
Source: PLoS One. 2025 Nov 20;20(11):e0335591. doi: 10.1371/journal.pone.0335591 (PMC12633885; doi:10.1371/journal.pone.0335591)
Supplement: S4 File — (PDF) [file pone.0335591.s004.pdf]

Fig 1. SPIRIT schedule of enrolment, interventions, and assessments for the CASSIOPEIA study.

	STUDY PERIOD							
	**Enrolment**	**Surgery**	**Post-surgery**					**Close-out**
**TIMEPOINT**	$-t_1$	**0**	t_1	t_2	t_3	t_4	t_5	t_6
**ENROLMENT:**	X	date of surgery	4 weeks	12 weeks	24 weeks	36 weeks	48 weeks	
**Eligibility screen**	X							
**Informed consent**	X							
**Allocation**	N/A							
**INTERVENTIONS:**	N/A							
**ASSESSMENTS:**								
Patient Characteristics	X							X
Tumor marker	X		X	X	X	X	X	
Total colonoscopy	X							
CT scan	X			X	X	X	X	
Blood collection for ctDNA		X	X	X	X	X	X	
Survival analysis								X
